# *Arabidopsis* MKK10-MPK6 mediates red-light-regulated opening of seedling cotyledons through phosphorylation of PIF3

**DOI:** 10.1093/jxb/erx418

**Published:** 2017-12-13

**Authors:** Xiaoyun Xin, Wenhao Chen, Bo Wang, Fan Zhu, Yuan Li, Hailian Yang, Jigang Li, Dongtao Ren

**Affiliations:** 1 State Key Laboratory of Plant Physiology and Biochemistry, College of Biological Sciences, China Agricultural University, China; 2 Collaborative Innovation Center of Crop Stress Biology, China

**Keywords:** *Arabidopsis thaliana*, cotyledon opening, MKK10-MPK6, phytochrome-interacting factor, phosphorylation, photomorphogenesis

## Abstract

Photomorphogenesis is an important process in which seedlings emerge from soil and begin autotrophic growth. Mechanisms of photomorphogenesis include light signal perception, signal transduction, and the modulation of expression of light-responsive genes, ultimately leading to cellular and developmental changes. Phytochrome-interacting factors (PIFs) play negative regulatory roles in photomorphogenesis. Light-induced activation of phytochromes triggers rapid phosphorylation and degradation of PIFs, but the kinases responsible for the phosphorylation of PIFs are largely unknown. Here, we show that Arabidopsis MPK6 is a kinase involved in phosphorylating PIF3 and regulating red light-induced cotyledon opening, a crucial process during seedling photomorphogenesis. MPK6 was activated by red light, and the cotyledon opening angle in red light was reduced in *mpk6* seedlings. MKK10, a MAPKK whose function is currently unclear, appears to act as a kinase upstream of MPK6 in regulating cotyledon opening. Activation of MPK6 by MKK10 led to the phosphorylation of PIF3 and accelerated its turnover in transgenic seedlings. Accordingly, the overexpression of PIF3 suppressed MKK10-induced cotyledon opening. MKK10 and MPK6 function downstream of phyB in regulating seedling cotyledon opening in red light. Therefore, the MKK10-MPK6 cascade appears to mediate the regulation of red-light-controlled seedling photomorphogenesis via a mechanism that might involve the phosphorylation of PIF3.

## Teaser

The kinases responsible for phosphorylation of phytochrome-interacting factors (PIFs) in plants undergoing photomorphogenesis are largely unknown. We show that the MKK10-MPK6 cascade regulates red light-induced photomorphogenesis through phosphorylation of PIF3.

## Introduction

Light is the primary energy source and an important signal for plant growth, development, and stress responses. The wavelength, intensity, duration, and direction of light are sensed by plants through a series of photoreceptors, including phytochromes (Li *et al.*, 2011), cryptochromes (Chaves *et al.*, 2011), phototropins (Christie, 2007), LOV/F-Box/Kelch repeat proteins (Ito *et al.*, 2012), and UV-B receptors (Jenkins, 2014). The signals from these photoreceptors are then transduced through distinct signaling pathways and influence many developmental and physiological processes throughout the plant’s life cycle, such as seed germination, seedling photomorphogenesis, shade avoidance, circadian rhythms, and flowering time.

Phytochromes are red (R) and far-red (FR) light photoreceptors in plants (Franklin and Quail, 2010; Li *et al.*, 2011) that are present in the inactive red-light-absorbing form (Pr) in darkness and convert to the active far-red-absorbing form (Pfr) upon exposure to R light (Fankhauser, 2001; Quail, 1997). The translocation of active phytochromes from the cytosol to the nucleus is a key event in the phytochrome signaling pathway (Kevei *et al.*, 2007; Nagatani, 2004). The *Arabidopsis thaliana* genome encodes five phytochromes, namely, phyA to phyE, which play overlapping but distinct roles in various responses to light (Bae and Choi, 2008). After germination, seedlings undergo skotomorphogenesis (etiolation) in darkness and photomorphogenesis (de-etiolation) in light. Skotomorphogenic phenotypes include elongated hypocotyls, exaggerated apical hooks, unopened cotyledons, and the development of the proplastids into etioplasts, whereas photomorphogenic phenotypes include inhibited hypocotyl elongation, opened and expanded cotyledons, and the development of chloroplasts (Arsovski *et al.*, 2012; Li *et al.*, 2011; Seluzicki *et al.*, 2017). FR-light-promoted seedling photomorphogenesis is primarily under the control of phyA, whereas R-light-induced photomorphogenesis is predominantly controlled by phyB (McCormac *et al.*, 1993; Parks and Quail, 1993; Reed *et al.*, 1994).

Phytochromes regulate light responses through downstream intermediates (Chen and Chory, 2011; Li *et al.*, 2011; Quail, 2010; Xu *et al.*, 2015). Phytochrome-interacting factors (PIFs), a subfamily of basic helix-loop-helix (bHLH) transcription factors, are central players in phytochrome-mediated light signaling networks that regulate the expression of over 1000 light-responsive genes (Castillon *et al.*, 2007; Leivar and Quail, 2011). In darkness, the inactivation of phytochromes allows PIFs to accumulate and promote skotomorphogenesis, whereas upon exposure to light, the activation of phytochromes induces phosphorylation and degradation of PIFs to initiate photomorphogenesis (Leivar and Monte, 2014; Leivar and Quail, 2011). In Arabidopsis, PIF1 is phosphorylated by Casein Kinase 2 (CK2), whereas PIF4 and PIF3 are phosphorylated by Brassinosteroid Insensitive 2 (BIN2) (Bernardo-García *et al.*, 2014; Bu *et al.*, 2011; Ling *et al.*, 2017); however, mutated PIF1 and PIF4 proteins in which the known phosphorylation sites were substituted by alanine residues continued to display robust light-induced phosphorylation. The kinase AsphyA phosphorylates PIF1, PIF3, and PIF4 in oat (*Avena sativa*); however, transgenic plants expressing mutant AsphyA protein with significantly lower kinase activity continued to show light-induced phosphorylation of PIF3 (Shin *et al.*, 2016). Photoregulatory protein kinases (PPKs), a small family of CK1-like proteins, were recently shown to phosphorylate PIF3; however, knockout of multiple PPK genes reduced but did not abolish light-induced PIF3 phosphorylation and degradation (Ni *et al.*, 2017). Over 20 phosphorylation sites have been identified in Arabidopsis PIF3 (Ni *et al.*, 2013), suggesting that other kinase(s) might also mediate the phosphorylation of PIFs and PIF-regulated light responses (Xu *et al.*, 2015).

Mitogen-activated protein kinase (MAPK) cascades are highly conserved signaling modules in eukaryotic cells composed of three types of kinases that are sequentially activated by phosphorylation: MAPKK kinases (MAPKKKs), MAPK kinases (MAPKKs), and MAPKs (MAPK Group, 2002). MAPK cascades are activated by upstream kinase(s) or receptor(s). Active MAPK phosphorylates a variety of substrate proteins, thereby altering their activity, localization, stability, and transcriptional level (Xu and Zhang, 2015). MAPK cascades in plants play important roles in regulating growth, development, and stress responses. Although the role of MAPK cascades in regulating plant light responses is far from clear, several lines of evidence suggest that they are involved in this process: two MAPKs in cucumber (*Cucumis sativus*) cotyledons are activated during FR- and R-light-induced seedling de-etiolation (Alvarez-Flórez *et al.*, 2013); the transcription of several MAPK cascade genes in Arabidopsis is regulated by light, and among these, *MKKK14* is up-regulated by R light and its null mutant shows a short-hypocotyl phenotype in FR- but not R-light-induced seedling de-etiolation (Khanna *et al.*, 2006; López-Juez *et al.*, 2008; Tepperman *et al.*, 2006); and the Arabidopsis MKK3-MPK6 cascade is activated by blue (B) light and regulates hypocotyl growth through phosphorylation of MYC2 (Sethi *et al.*, 2014). Arabidopsis contains 60 MAPKKKs, 10 MAPKKs, and 20 MAPKs (nominated as MKKKs, MKKs, and MPKs, respectively) (MAPK Group, 2002). MKKs function in the following processes: MKK1 in defense, abscisic acid, and reactive oxygen species responses (Pitzschke *et al.*, 2009; Qiu *et al.*, 2008; Xing *et al.*, 2008); MKK2 in cold and salt tolerance (Teige *et al.*, 2004), MKK3 in hypocotyl growth and jasmonic acid responses (Sethi *et al.*, 2014; Takahashi *et al.*, 2007); MKK4/MKK5 in H_2_O_2_ (Ren *et al.*, 2002) and NO production (Wang *et al.*, 2010*a*), stomatal and ovule development (Wang *et al.*, 2008; Wang *et al.*, 2007), and defense responses (Asai *et al.*, 2002; Wang *et al.*, 2010*b*); MKK6 in cytokinesis (Soyano *et al.*, 2003); MKK7 in polar auxin transport (Dai *et al.*, 2006; Jia *et al.*, 2016); and MKK4/MKK5/MKK9 in ethylene and camalexin biosynthesis (Ren *et al.*, 2008; Xu *et al.*, 2008). However, the activities and biological functions of MKK8 and MKK10 remain obscure.

In this study, we show that MPK6 is activated by R light. MPK6 loss-of-function mutant seedlings displayed less pronounced cotyledon opening in R light than did wild-type seedlings. MKK10 functions upstream of MPK6 in regulating cotyledon opening. After MKK10-mediated activation of MPK6, this latter kinase phosphorylated PIF3 *in vitro* and *in vivo*. Phosphorylation of PIF3 by MKK10-MPK6 accelerated the turnover of PIF3 in transgenic seedlings in darkness. Overexpression of PIF3 suppressed MKK10-induced cotyledon opening in dark-grown seedlings. Genetic analysis demonstrated that the MKK10-MPK6 cascade functions downstream of phyB in regulating cotyledon opening in seedlings exposed to R light. Our findings suggest that MKK10-MPK6-PIF3 is a module that regulates phyB-mediated seedling photomorphogenesis in R light.

## Materials and methods

### Plant materials

Seeds of *Arabidopsis thaliana* Col-0 wild type, mutants, and transgenic lines were surface sterilized, incubated in the dark for 4 days at 4 °C, and sown on 0.8% agar plates containing 0.5×Murashige and Skoog (MS) medium, pH 5.7, and 1% sucrose. Seven-day-old seedlings were transferred from the plates to soil and grown at 22 °C in a growth room under a 16 h light/8 h dark photoperiod at a photon flux density of 100 μmol m ^–2^ s^–1^. To analyze seedling phenotypes, seeds were treated at 4 °C and sown on 0.8% agar plates containing 1×MS, pH 5.7. After exposure to 100 μmol m^–2^ s^–1^ white light for 3 h, plates were transferred to darkness, R (1, 8, or 30 μmol m^–2^ s^–1^), FR (30 μmol m^–2^ s^–1^), or B (8 μmol m^–2^ s^–1^) light conditions and grown for 4 days. To induce expression of the transgenes 0.02 or 0.05 μmol dexamethasone (DEX) was added to the medium.

### Vector construction

Total RNA was isolated from seedlings using Trizol reagent (Invitrogen). First-strand cDNA was synthesized with M-MLV virus reverse transcriptase (Promega) using dT(16) as the primer and RNA as the template.

The coding regions of *MKKs* and *MPKs* with an *Nde*I site added before the first ATG codon were obtained by PCR and cloned into a *p*Bluescript vector. Point mutations were introduced into the *MKKs* with a Quick-Change Site-directed Mutagenesis Kit (Stratagene). The *Nde*I/*Xho*I fragments of *MKKs* were cloned into a modified *p*Bluescript vector with an Ω sequence and Flag-epitope tag coding sequence at the 5ʹ end (Xu *et al.*, 2008). *Spe*I/*Xho*I fragments of *MKKs* were ligated into a *p*TA7002 vector (Aoyama and Chua, 1997). A 2.5 kb fragment before the first ATG codon of *MKK10* was amplified by PCR, using genomic DNA as the template, and used as the *MKK10* native promoter. The promoter fragment was inserted into a *p*CAMBIA 1300-221 vector (Invitrogen) to substitute the CaMV35S promoter sequence. The PIF3 coding region with the stop codon removed was added before the Green Fluorescent Protein (GFP) sequence in the modified *p*CAMBIA 1300 vector; the MPK6-mCherry coding sequence was inserted into the modified *p*CAMBIA 1300 vector to substitute for the GFP coding sequence (Cao *et al.*, 2010). The resulting constructs were transformed into *Agrobacterium tumefaciens* strain GV3101.

The coding regions of *PIF3* with an *Nde*I site added before the first ATG codon and an *Xho*I site added to its 3ʹ end were obtained by PCR and cloned into a *p*Bluescript vector. The *Nde*I/*Xho*I fragment of *PIF3* and the *Nde*I/*Sal*I fragment of *MKK10* mutants were cloned into a *p*GEX4T-2 vector. The *Nde*I/*Sal*I fragments of *MPK3* and *MPK6*, and *Bam*HI/*Sal*I fragments of *MPK12* and *MPK10* were cloned into a *p*ET28a(+) vector. The resulting constructs were transformed into *Escherichia coli* strain BL21.

Primers used are listed in Table S1 available at the Dryad Digital Repository, http://dx.doi.org/10.5061/dryad.hq7b8.

### *Agrobacterium*-mediated transformation

Transgenic Arabidopsis plants were generated using the floral dip method (Clough and Bent, 1998). *MKKs* and *MKK10* promoter-*GUS* transgenic plants were screened with 15 mg/l hygromycin, and expression of the transgene was detected using an immunoblot analysis and β-glucuronidase (GUS) staining.

Transient transformation of tobacco (*Nicotiana benthamiana*) leaves was performed as described previously (Xu *et al.*, 2008). PIF3-GFP and MPK6-mCherry fluorescence signals were viewed under a Zeiss LSM510 Meta Confocal Laser-Scanning System.

### Mutant generation and genetic crosses

To generate the *mkk10* mutant, an MKK10-specific target for Cas9 was selected to mutate *MKK10* using CRISPR-PLANT (http://www.genome.arizona.edu/crispr/CRISPRsearch.html). The target sequence was cloned into a CRISPR/Cas9 vector, *p*HEE 401, as described previously (Wang *et al.*, 2015). The resulting construct was transformed into *A. tumefaciens* strain GV3101. Arabidopsis transformation was performed using the floral dip method (Clough and Bent, 1998). Transgenic seedlings were screened using hygromycin selection, genomic PCR, and sequencing as described previously (Wang *et al.*, 2015). The *mkk10* mutant obtained had two insertions, an A after base 136 and a T after base 386 of the start codon in the *MKK10* gene. An *Mlu*I site in the MKK10 genomic sequence was mutated in *mkk10* due to the insertion of a T base, and was used for *mkk10* mutant genotyping.

T-DNA insertional mutants, including *pif3* (Salk_030753), *mpk3* (SALK_100651), *mpk6-3* (SALK_127507), *mpk6-4* (SALK_062471), and *mkk9* (SAIL_060_H06), were obtained from the Arabidopsis Biological Resource Center. The homozygous mutants were screened using genomic PCR and reverse transcription (RT)-PCR. *phyA* (*phyA-211*) and *phyB* (*phyB-9*) were the point mutation mutants. *cry1* (*cry1-304*) was a fast-neutron mutation mutant. *PIF3 OE* was a gift from Dr Giltsu Choi (Park *et al.*, 2004). The homozygous mutants were identified as described previously (Mockler *et al.*, 1999; Reed *et al.*, 1993; Reed *et al.*, 1994).

Genetic crossing was performed to generate *MKK10*^*D*^/*PIF3 OE*, *MKK10*^*D*^/*mpk6*, *MKK10*^*D*^/*phyA*, *MKK10*^*D*^/*phyB*, *MKK10*^*D*^/*cry1*, *mkk9/mkk10*, and *mpk6*/*pif3* mutants. The *mpk6-4* mutant was used for the kinase activity assay and for genetic crossing.

### Preparation of recombinant proteins

*Escherichia coli* cells transformed with *p*GEX4T-2 or *p*ET28a(+) constructs were cultured, and isopropyl-D-thiogalactopyranoside was added to induce the recombinant protein expression. His-MPK proteins were purified using a Ni^2+^-Cheating Sepharose Fast Flow column (GE Healthcare), and GST-PIF3 or GST-MKK10 mutant proteins were purified using a Glutathione Sepharose 4B column (GE Healthcare).

### Protein extraction and immunoblot analysis

Proteins were extracted from seedlings using kinase buffer as previously described (Xu *et al.*, 2008) and used for in-gel kinase assays and immunoblot analysis of the MPKs, MKKs (except for MKK7), and tubulin. For the MKK7 expression assay, proteins were extracted using 1×SDS sample buffer (diluted with kinase pre-mix buffer: 100 mM HEPES, pH 7.5, 5 mM EDTA, 5 mM EGTA, 10 mM Na_3_VO_4_, 10 mM NaF, 50 mM β-glycerophosphate). For the MYC-PIF3 expression assay, proteins were extracted using kinase buffer containing 40 μM MG132 for co-immunoprecipitation (Co-IP) and boiling extraction buffer for Phos-tag mobility shift assays (Al Sady *et al.*, 2006). Protein separation and immunoblotting were performed as described previously (Xu *et al.*, 2008). The primary antibodies included anti-Flag, anti-MYC, anti-MPK3, anti-MPK6, and anti-β-tubulin antibodies. Secondary antibodies included horseradish peroxidase-conjugated goat anti-mouse or anti-rabbit antibodies.

### Co-immunoprecipitation assay

For the MYC-PIF3 and MPK6 Co-IP assay, protein extracts were incubated with anti-MYC beads for 3.5 h at 4 °C in the dark on a rotating mixer. The beads were collected and washed four times with kinase pre-mix buffer containing 0.02% Triton X-100. Bound proteins were eluted in 1 M NH_3_OH, pH 11.5, with 5% SDS, and separated by 10% SDS-PAGE. Immunoblot assays were performed using anti-MPK6 and anti-MYC antibodies as the primary antibodies.

### Pull-down assay

GST or GST-PIF3 proteins were incubated with Glutathione Sepharose 4B beads pre-balanced with extraction buffer (0.1 M Tris-HCl, pH 7.5, 150 mM NaCl) at 4 °C for 1 h. The beads were collected, washed three times with washing buffer (0.1 M Tris-HCl, pH 7.5, 150 mM NaCl, 0.02% Triton X-100), and incubated with His-MPK6 protein in binding buffer (50 mM Tris-HCl, pH 7.5, 100 mM NaCl, 0.02% Triton X-100) at 4 °C for 2 h. After centrifugation, the beads were washed five times with washing buffer. The beads were boiled in 1×SDS buffer, and the proteins were separated by 10% SDS-PAGE. Immunoblot assays were performed using anti-GST and anti-His antibodies as the primary antibodies.

### Kinase assays

GST-MKK10 mutant proteins were incubated with His-MPKs in phosphorylation buffer (20 mM Tris-HCl, pH 7.5, 10 mM MgCl_2_, and 50 µM ATP) at 30 °C for 30 min. A 0.5 µg sample of His-MPK was taken from each reaction and added into a new tube with 5 µg myelin basic protein (MBP) and 1 μCi [γ-^32^P]-ATP in phosphorylation buffer. After incubation at 30 °C for 30 min, the reactions were stopped by adding 4×SDS buffer. The reaction mixture was heated, and the proteins were separated by 10% SDS-PAGE. The gels were dried, and phosphorylation of MBP was detected by autoradiography.

An in-gel kinase assay was performed as described previously (Ren *et al.*, 2002).

### Cotyledon opening assay in seedlings

Cotyledon opening angles were measured and analyzed using ImageJ software (http://rsb.info.nih.gov/ij/). Cotyledon angle represents the angle between the lines drawn through two cotyledons. An angle of 0° indicates fully folded cotyledons.

### Real-time quantitative RT-PCR

Real-time quantitative RT-PCR (Q-PCR) was performed using a SYBR Premix EX Taq^TM^ Kit (Takara) in the presence of SYBR Green I. Amplification was monitored in real time with a 7500 real-time PCR system (Applied Biosystems). The expression levels of selected genes were normalized to the *UBQ10* control. Primers used for Q-PCR are listed in Table S1 (available at the Dryad Digital Repository, http://dx.doi.org/10.5061/dryad.hq7b8.

### Accession number

The accession numbers of genes used in this study are listed in Table S2 at Dryad.

## Results

### MPK6 is activated by red light and mediates red-light-regulated cotyledon opening

**Fig. 1.**
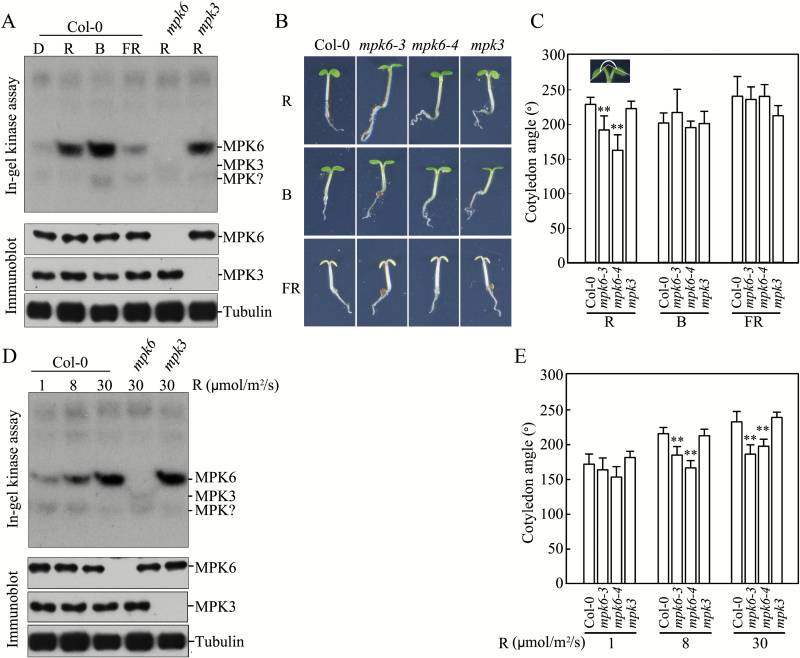
MPK6 is activated by different light conditions and loss of MPK6 activity inhibits cotyledon opening in seedlings in R light. (A) Kinase activity in Col-0, *mpk3*, and *mpk6* seedlings detected in an in-gel kinase assay. MPK3, MPK6, and tubulin were detected by immunoblot analysis. (B) Photographs of Col-0, *mpk3*, and *mpk6* seedlings showing the cotyledon phenotypes. White bar=2 mm. (C) Cotyledon opening angles in Col-0, *mpk3*, and *mpk6* seedlings in R, B, and FR light. (D) Kinase activity in Col-0 seedlings grown in different R light conditions detected in an in-gel kinase assay. (E) Cotyledon opening angles of Col-0, *mpk3*, and *mpk6* seedlings in different R light conditions. Data are means±SD (*n*=30). Asterisks indicate significant differences between Col-0 and *mpk6* seedlings (Student’s *t*-test, **P<0.01). D, dark; R, red light; FR, far-red light; B, blue light.

MPK6 is activated in seedlings transferred from darkness to B light, and *mpk6* mutant seedlings have shorter hypocotyls than wild-type seedlings in white, R, FR, and B light (Sethi *et al.*, 2014). Therefore, the activation of a MAPK cascade might mediate the signaling pathway underlying inhibited hypocotyl elongation, an important aspect of seedling photomorphogenesis. To explore whether a MAPK cascade (and, if so, which one) is involved in regulating the cotyledon opening process, another crucial aspect of seedling photomorphogenesis, we analyzed MAPK activity and cotyledon opening in seedlings grown in R, FR, and B light. MAPK activity in total proteins extracted from seedlings grown in darkness, 8 μmol m^–2^ s^–1^ of R light, 30 μmol m^–2^ s^–1^ of FR light, or 8 μmol m^–2^ s^–1^ of B light for 4 days was detected using in-gel kinase activity assays, with MBP as an artificial MAPK substrate. The extract from wild-type (Col-0) seedlings grown in the dark contained three kinases with lower basal-level activity (with molecular weights of ~ 60, 46, and 40 kD) (Fig. 1A). However, the activity of the 46 kD kinase increased strongly in seedlings grown in R and B light, and slightly in seedlings grown in FR light, compared with dark-grown seedlings. The activity of the 40 kD kinase was slightly decreased in seedlings grown in R light and increased in seedlings grown in B and FR light compared with seedlings grown in the dark. Finally, the activity of the 60 kD kinase did not significantly differ among seedlings grown in the dark or in R, B, or FR light. We also analyzed kinase activity using extracts from seedlings of *MPK3* and *MPK6* T-DNA insertional null mutants. The lack of MPK3 protein in *mpk3* seedlings and MPK6 protein in *mpk6* seedlings was confirmed by immunoblot analysis with MPK3- or MPK6-specific antibodies. The 46 kD kinase was not activated in *mpk6* seedlings grown in R light, indicating that this kinase was MPK6 (Fig. 1A).

Since R, FR, and B light activated MPK6 in seedlings, we compared the hypocotyl lengths and cotyledon opening angles of Col-0, *mpk6*, and *mpk3* seedlings grown under these different light conditions. We used two alleles of *mpk6* (*mpk6-3* and *mpk6-4*) to exclude any possible effects of an additional mutation in the *mpk6* mutants. As shown in Fig. 1B and C, the cotyledon opening angles of *mpk6-3* and *mpk6-4* seedlings grown in R light were 16% and 28% lower, respectively, than those of Col-0 seedlings, whereas no significant differences were observed in FR or B light. Under all light conditions, the cotyledon opening angles of *mpk3* seedlings did not significantly differ from those of Col-0 seedlings. In addition, the hypocotyl lengths of Col-0, *mpk6*, and *mpk3* seedlings did not differ significantly under darkness or under the different light conditions (Fig. S1 at Dryad). These results suggest that the activation of MPK6 by R light might play a role in R-light-induced cotyledon opening.

To confirm the R-light-dependent activation of MPK6 and to explore the role of MPK6 in regulating cotyledon opening, we analyzed MAPK activity and cotyledon-opening angles in seedlings grown under different fluence rates of R light (1, 8, and 30 μmol m^–2^ s^–1^). In wild-type seedlings, MPK6 activity increased in R light in an intensity-dependent manner (Fig. 1D). The cotyledon opening angles of *mpk6-3* and *mpk6-4* seedlings were significantly reduced when grown in 8 and 30 μmol m^–2^ s^–1^ R light compared with Col-0 seedlings, but these angles were not significantly different in 1 μmol m^–2^ s^–1^ R light. The cotyledon opening angles of *mpk6-3* and *mpk6-4* seedlings were 14% and 23% lower in 8 μmol m^–2^ s^–1^ R light and 20% and 15% lower in 30 μmol m^–2^ s^–1^ R light, respectively, than those of Col-0 seedlings (Fig. 1E). The cotyledon opening angles of Col-0 and *mpk3* seedlings grown under the same fluence rate of R light did not differ significantly. These results suggest that a MAPK cascade involving MPK6 mediates the R-light-regulated seedling photomorphogenesis signaling pathway.

### MKK10 is the predominant Arabidopsis MAPKK that regulates cotyledons opening

Since the activation of MPK6 is involved in regulating cotyledon opening in seedlings, we speculated that the activation of an MKK upstream of MPK6 might cause the same phenotype. To support this hypothesis, we generated transgenic kinase-active Arabidopsis lines for various MKKs and used them to identify MAPKK(s) that function upstream of MPK6 in regulating seedling cotyledon opening. Based on information from previous reports (Jia *et al.*, 2016; Matsuoka *et al.*, 2002; Ren *et al.*, 2002; Takahashi *et al.*, 2007; Teige *et al.*, 2004; Xu *et al.*, 2008) and sequence analyses, we generated kinase-active lines for all 10 MKKs in Arabidopsis. For MKK1 to MKK9, the Thr (T) and Ser (S) residues in the T/SxxxxxS/T motif in the activation loops were substituted with Asp (D) (designated as MKK1^DD^ to MKK9^DD^). Owing to the absence of the typical T/SxxxxxS/T motif in the MKK10 activation loop, and since no MKK10-active mutant has been reported previously, we substituted Ser197 in its putative activation domain with Asp (D) to generate MKK10^D^, a constitutive mutant for MKK10 (Fig. S2A at Dryad). The mutated *MKK1* to *MKK10* genes were placed under the control of the DEX-inducible promoter in the *p*TA7002 vector (Aoyama and Chua, 1997) and transformed into Arabidopsis. For *MKK3*^*DD*^, over 20 transgenic lines showing hygromycin resistance were obtained; unfortunately, MKK3^DD^ protein expression was not detected in any of these lines in an immunoblot analysis. In contrast to *MKK3*^*DD*^, we obtained transgenic plants with normal induction of MKK10^D^ protein for *MKK10*^*D*^ and with normal induction of MKKx^DD^ proteins for the eight *MKKx*^*DD*^ genes.

**Fig. 2.**
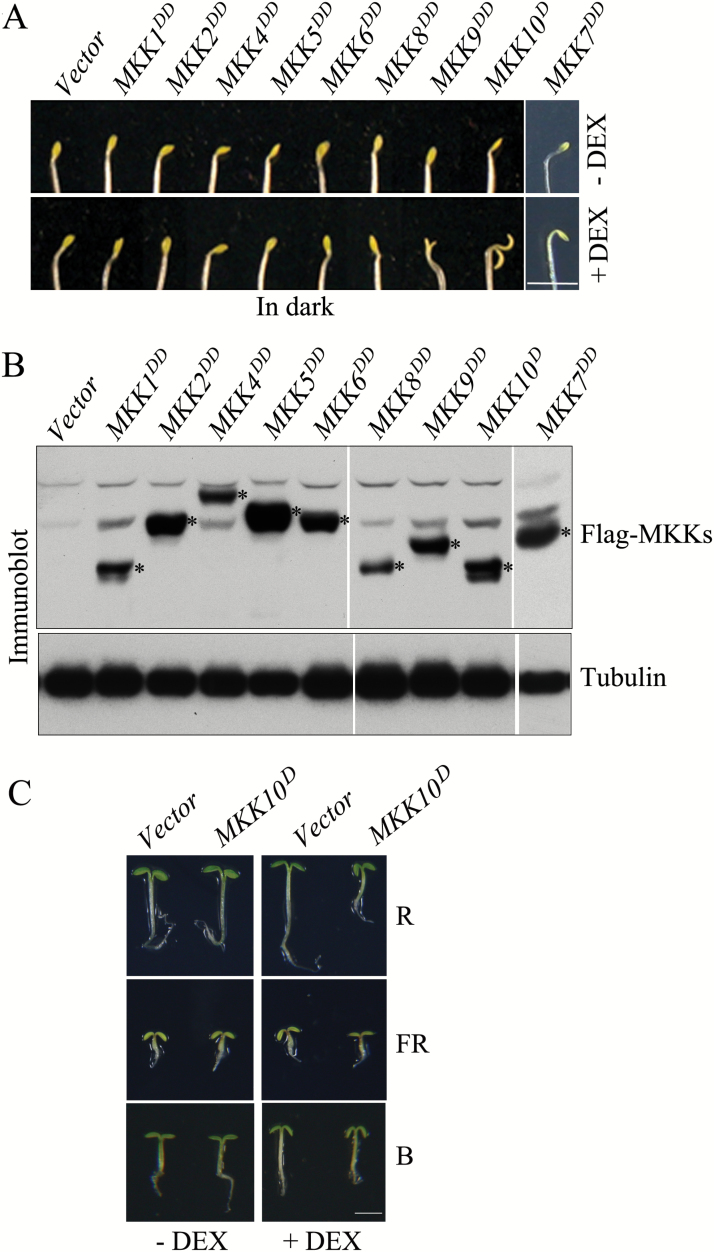
Analysis of cotyledon opening in *MKK*s active mutant transgenic seedlings. (A) Photographs showing cotyledon opening phenotypes in *MKK*s transgenic seedlings with (+DEX) or without (–DEX) induction of transgenes. Scale bar=2 mm. (B) Protein levels of MKKs in transgenic seedlings detected by immunoblot analysis with anti-Flag antibody. Asterisks indicate Flag-MKK proteins. (C) Cotyledon opening phenotypes in *MKK10*^*D*^ and *Vector* transgenic seedlings in R, FR, and B light with (+DEX) or without (–DEX) induction of transgenes.

Homozygous *MKKx*^*DD*^ and *MKK10*^*D*^ transgenic seeds were germinated and grown on plates with or without DEX in dark. Uninduced (–DEX) *MKKs* transgenic seedlings did not exhibit cotyledon opening in dark, and nor did induced (+DEX) *MKK1*^*DD*^ to *MKK8*^*DD*^ seedlings. However, *MKK10*^*D*^ seedlings had large cotyledon opening angles, and *MKK9*^*DD*^ seedlings had slightly open cotyledons after MKK10^D^ or MKK9^DD^ protein induction (+DEX) in the dark. When germinated and grown under light conditions, the induction of MKK10^D^ led to seedlings with large cotyledon opening angles in both R and B light, whereas FR light did not affect cotyledon opening angles compared with the *Vector* control. All of the *MKKs* transgenic seedlings expressed the transgenes effectively, as shown by immunoblot analysis (Fig. 2). These results suggest that MKK10 is the MKK that plays a major role in regulating cotyledon opening and that MKK9 may function redundantly with MKK10 in this process.

**Fig. 3.**
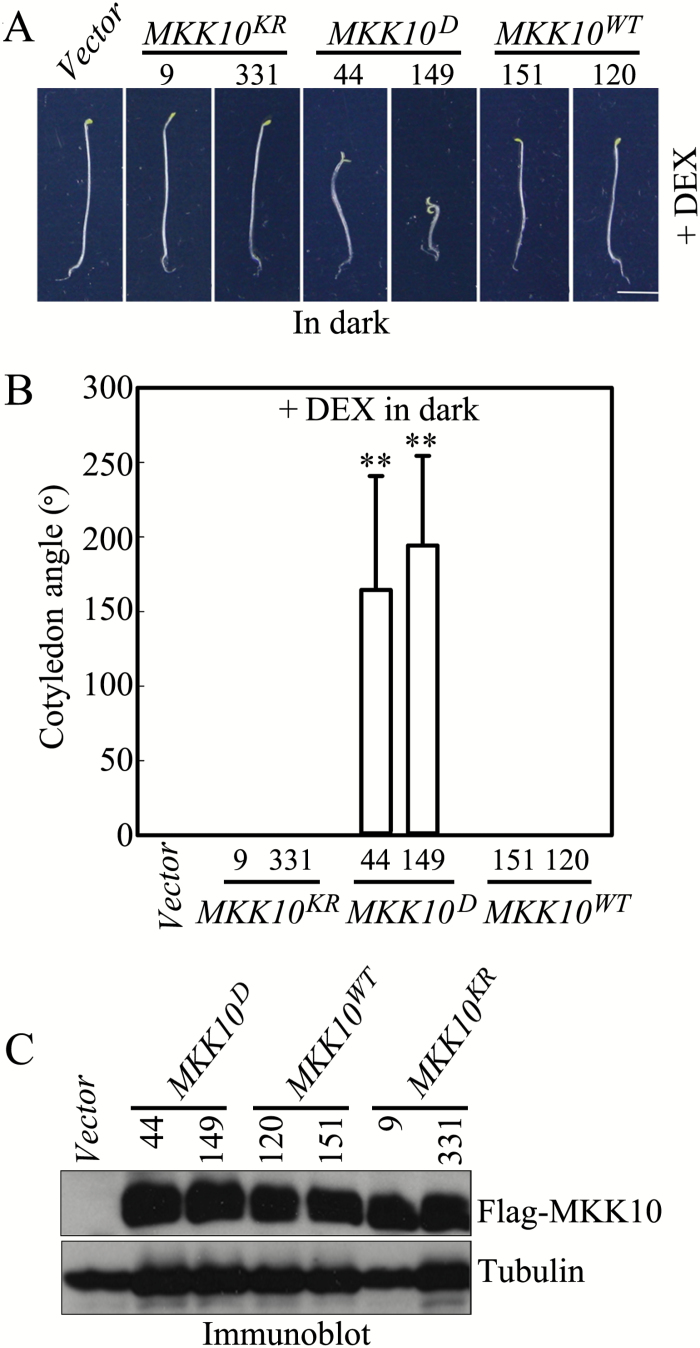
Expression of active mutant MKK10 induces cotyledon opening in seedlings. (A) Photographs showing cotyledon opening phenotypes in *MKK10*^*WT*^, *MKK10*^*D*^, and *MKK10*^*KR*^ seedlings. Scale bar=2 mm. (B) Cotyledon opening angles in transgenic seedlings. Data are means±SD (*n*=30). Asterisks indicate significant differences between *MKK10*^*D*^ and *Vector* transgenic seedlings (Student’s *t*-test, **P<0.01). (C) Protein levels of MKK10^WT^, MKK10^D^, and MKK10^KR^ in transgenic seedlings detected by immunoblot analysis with anti-Flag antibody.

To confirm the importance of MKK10 activity in inducing cotyledon opening, we generated MKK10^KR^, a kinase-inactive form of MKK10 with the Lys77 (K) in its ATP-binding site substituted with Arg (R) (Fig. S2B at Dryad). We generated *MKK10*^*KR*^ and *MKK10*^*WT*^ transgenic plants and analyzed independent transgenic lines for *MKK10*^*D*^, *MKK10*^*KR*^, and *MKK10*^*WT*^ to exclude any possible effects of the transgene insertion on phenotype. Consistent with the results shown in Fig. 2A, both *MKK10*^*D*^ lines displayed markedly increased cotyledon opening after transgene induction in the dark, but the *Vector*, *MKK10*^*KR*^, and *MKK10*^*WT*^ seedlings did not (Fig. 3A). Statistical analyses revealed that cotyledon opening angles in both *MKK10*^*D*^ lines were significantly larger than those in the *Vector*, *MKK10*^*KR*^, and *MKK10*^*WT*^ lines (Fig. 3B). Immunoblot analysis showed that all MKK10 variant transgenic lines expressed comparable levels of Flag-MKK10 protein (Fig. 3C). These results suggest that only the expression of the MKK10 active form caused cotyledons to open in darkness. The *MKK10*^*D*^ lines also had shortened hypocotyls, and in both lines, the hypocotyl length was negatively correlated with MPK6 activity (Figs 2C, 3A, and S3 at Dryad). To explore where MKK10 normally functions, we expressed *GUS* under the control of the native *MKK10* promoter in seedlings of both independent transgenic lines to examine the expression pattern of *MKK10*. GUS activity staining was detected only in the cotyledons of seedlings and not in hypocotyls (Fig. S4 at Dryad). This result implies that MKK10 normally regulates cotyledon opening but not hypocotyl length at the seedling stage. Perhaps the inhibited hypocotyl elongation in *MKK10*^*D*^ seedlings is due to the strong expression *MKK10*^*D*^ in overexpression lines.

**Fig. 4.**
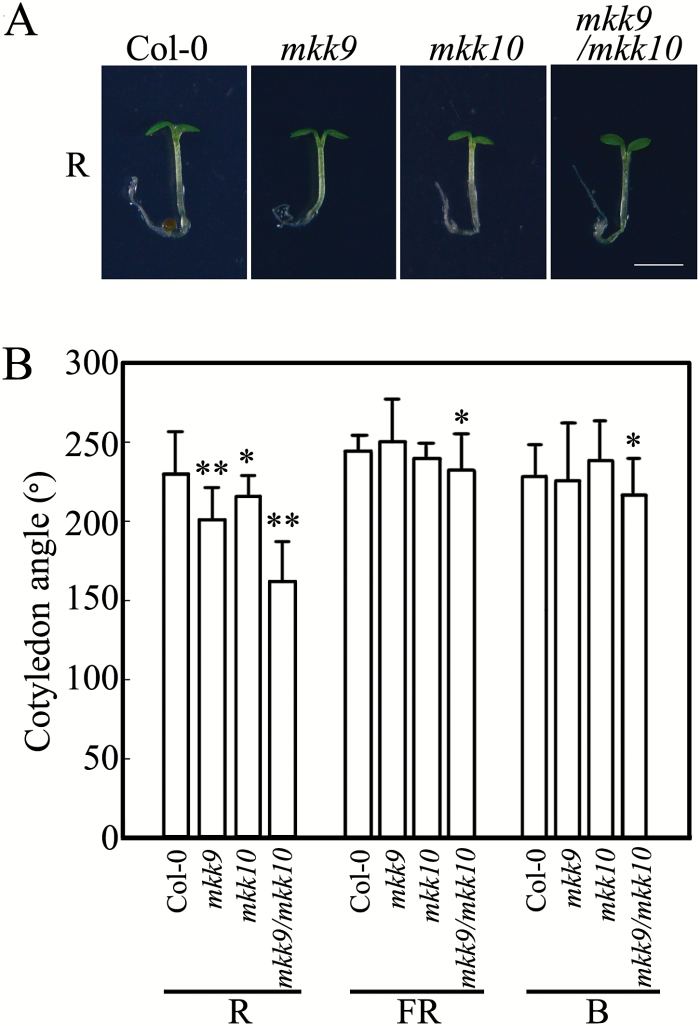
Loss of function of *MKK9* and *MKK10* reduces R-light-induced cotyledon opening angles in seedlings. (A) Photographs of Col-0, *mkk9*, *mkk10*, and *mkk9/mkk10* seedlings showing cotyledon phenotypes. Scale bar=2 mm. (B) Cotyledon opening angles in Col-0, *mkk9*, *mkk10*, and *mkk9/mkk10* seedlings in R, FR, and B light. Data are means±SD (*n*=30). Asterisks indicate significant differences between Col-0 and mutant seedlings (Student’s *t*-test, *P<0.05, **P<0.01).

We examined the cotyledon opening phenotype in the *mkk10* and *mkk9* mutants. *mkk9* is a T-DNA insertional null mutant, and *mkk10* is a mutant that we generated in this study using the CRISPR/Cas9 method (Fig. S5 at Dryad). Sequence analysis revealed two single nucleotide insertions in the coding region of *MKK10* in *mkk10*, resulting in an early stop codon in *MKK10* mRNA. As shown in Fig. 4A and B, the cotyledon opening angles of *mkk9* and *mkk10* seedlings were 12% and 6% lower than those of Col-0 wild-type seedlings, respectively, when grown in R light. However, the cotyledon opening angles of *mkk9*/*mkk10* double mutant seedlings were strongly reduced, to 30% lower than those of Col-0 seedlings. When grown in FR and B light, differences among the cotyledon opening angles of Col-0, *mkk9*, and *mkk10* seedlings were negligible, and the cotyledon opening angles of *mkk9/mkk10* seedlings were reduced by only 4% compared with Col-0 seedlings. These results suggest that *MKK10* and *MKK9* function redundantly in R-light-regulated cotyledon opening, which is consistent with the results of overexpression analysis.

### MKK10 functions upstream of MPK6 in regulating cotyledon opening

**Fig. 5.**
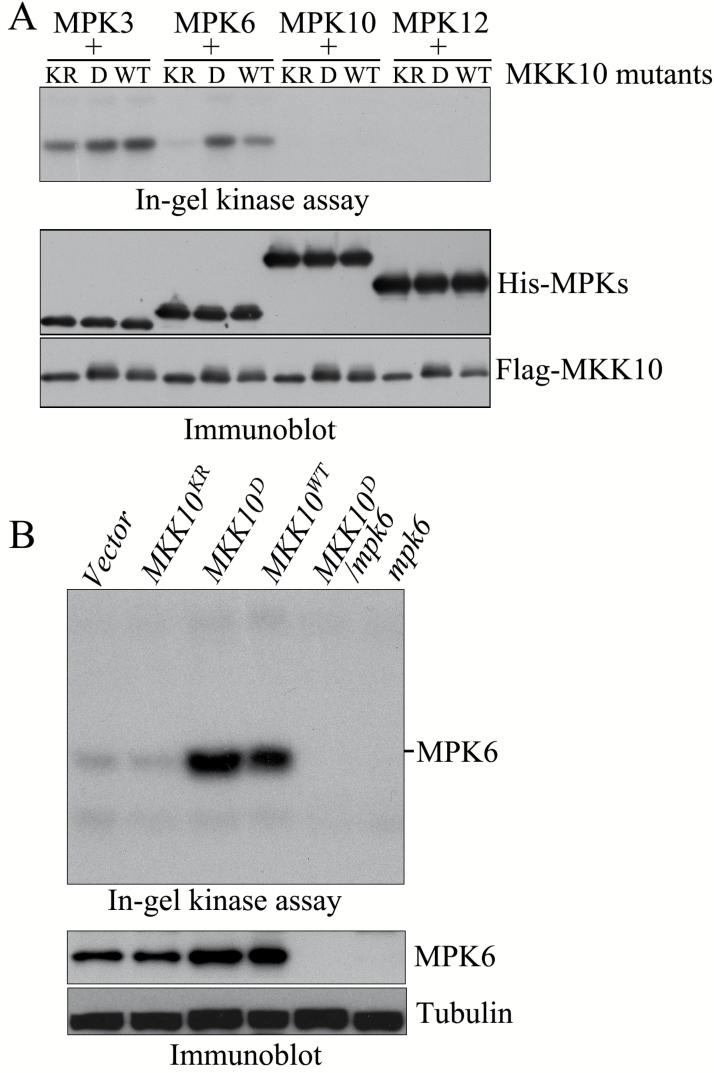
MKK10 activates MPK6 *in vitro* and *in vivo*. (A) Activation of His-MPKs by Flag-MKK10 mutant proteins *in vitro*. His-MPK activity was detected using an in-gel kinase assay (upper panel). Flag-MKK10 mutant proteins and His-MPK proteins in the reaction were detected by immunoblot analysis with anti-Flag and anti-His antibodies (lower panel). (B) MPK6 activity in *MKK10* mutant transgenic lines and in lines crossed with these plants detected by in-gel kinase assays (upper panel). MPK6 protein were detected by immunoblot analysis with anti-MPK6 antibody(lower panel).

We next performed a series of experiments to determine whether MKK10 is the MKK that functions upstream of MPK6. First, the recombinant MKK10 variant proteins were used to phosphorylate MPK6, and then MPK6 kinase activity was examined using MBP as substrate. MPK3, MPK10, and MPK12 were used as controls. As shown in Fig. 5A, MKK10^D^ strongly activated MPK6, while MKK10^WT^ moderately activated MPK6 and MKK10^KR^ did not activate this kinase. MPK10 and MPK12 were not activated by MKK10, but MPK3 was weakly activated by MKK10. We then generated transgenic Arabidopsis plants harboring *MKK10*^*WT*^, *MKK10*^*D*^, and *MKK10*^*KR*^ and subjected them to an in-gel kinase assay. As shown in Fig. 5B, after transgene induction, *MKK10*^*D*^ seedlings showed strong activation of a 46 kD kinase, *MKK10*^*WT*^ seedlings showed moderate activation of this kinase, and *MKK10*^*KR*^ and *Vector* seedlings showed only lower basal levels of 46 kD kinase activity. Therefore, following previously developed rules for the functions of MKKs (Jia *et al.*, 2016; Matsuoka *et al.*, 2002; Ren *et al.*, 2002; Takahashi *et al.*, 2007; Teige *et al.*, 2004; Xu *et al.*, 2008), MKK10^D^ is considered to be the active MKK10 and MKK10^KR^ the inactive MKK10. Third, to confirm that the 46 kD kinase activated by MKK10 in transgenic plants is MPK6, we generated a *MKK10*^*D*^/*mpk6* mutant by genetic crossing. An in-gel kinase assay showed that the activation of the 46 kD kinase by MKK10^D^ induction was abolished in *MKK10*^*D*^/*mpk6* seedlings, demonstrating that the 46 kD kinase is indeed MPK6 (Fig. 5B). These results suggest that MKK10 is an MKK that functions upstream of MPK6.

**Fig. 6.**
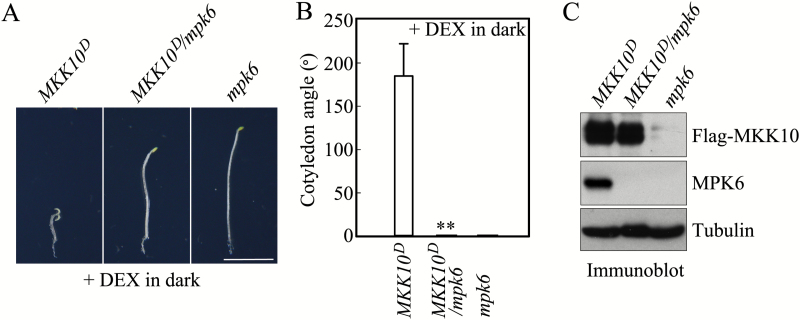
Activation of MPK6 by MKK10 is required for MKK10-regulated cotyledon opening in seedlings. (A) Photographs showing the cotyledon opening phenotypes in *MKK10*^*D*^, *MKK10*^*D*^*/mpk6*, and *mpk6* seedlings. Scale bar=2 mm. (B) Cotyledon opening angles in *MKK10*^*D*^, *MKK10*^*D*^*/mpk6*, and *mpk6* seedlings. Data are mean ±SD (*n*=30). Asterisks indicate significant differences between *MKK10*^*D*^ and *MKK10*^*D*^*/mpk6* seedlings (Student’s *t*-test, **P<0.01). (C) Protein levels of MKK10^D^ and MPK6 detected by immunoblot with anti-Flag and anti-MPK6 antibodies.

We analyzed cotyledon opening in *MKK10*^*D*^ and *MKK10*^*D*^/*mpk6* seedlings. As shown in Fig. 6A, after MKK10^D^ induction in the dark (+DEX in dark), *MKK10*^*D*^ seedlings displayed a strong cotyledon opening phenotype, whereas *MKK10*^*D*^/*mpk6* seedlings did not (Fig. 6B). Immunoblot analysis showed that comparable levels of MKK10^D^ were present in *MKK10*^*D*^ and *MKK10*^*D*^/*mpk6* seedlings (Fig. 6C). These results suggest that activation of MPK6 by MKK10 causes the cotyledon opening phenotype of *MKK10*^*D*^ seedlings.

### MKK10-MPK6 functions downstream of phyB in regulating cotyledon opening

**Fig. 7.**
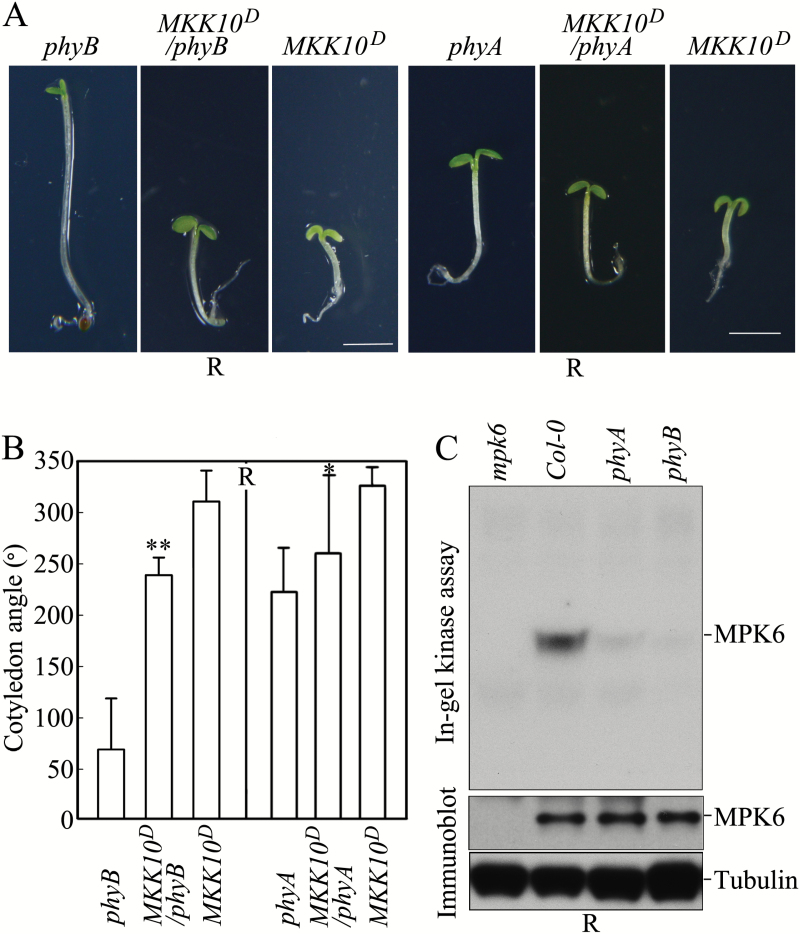
phyB functions upstream of MKK10-MPK6 in regulating cotyledon opening in seedlings in R light. (A) Photographs showing the cotyledon opening phenotype in *MKK10*^*D*^, *MKK10*^*D*^/*phyB*, and *MKK10*^*D*^/*phyA* seedlings in R light. Scale bar=2 mm. (B) Cotyledon opening angles in *MKK10*^*D*^, *MKK10*^*D*^/*phyB*, and *MKK10*^*D*^/*phyA* seedlings in R light. Data are means±SD (*n*=30). Asterisks indicate significant differences between *phyB* and *MKK10*^*D*^*/phyB* or between *phyA* and *MKK10*^*D*^/*phyA* (Student’s *t*-test, *P<0.05, **P<0.01). (C) Kinase activity in Col-0, *mpk6*, *phyA*, and *phyB* seedlings in R light detected using an in-gel kinase assay (upper pane;). MPK6 protein was detected by immunoblot analysis with anti-MPK6 antibody (lower panel).

The phytochrome phyB is the predominant phytochrome that regulates seedling photomorphogenesis in R light, and phyA has partially overlapping functions with phyB (Li *et al.*, 2011; Reed *et al.*, 1994). We therefore analyzed MPK6 activity in *phy* mutant seedlings grown in R light. As shown in Fig. 7, MPK6 was strongly activated in Col-0 seedlings grown in R light; however, R light only weakly activated MPK6 in the *phyA* mutant and did not activate MPK6 in the *phyB* mutant. We crossed *phyA* and *phyB* into the *MKK10*^*D*^ background. The reduced cotyledon opening angle in *phyB* mutant seedlings grown in R light was rescued (increased by 253%) in *MKK10*^*D*^/*phyB* after MKK10^D^ induction. By contrast, the cotyledon opening angle was only slightly (~17%) higher in *MKK10*^*D*^/*phyA* seedlings in R light compared with *phyA* mutant seedlings (Fig. 7A and B). The induction of MKK10^D^ in *MKK10*^*D*^/*cry1* seedlings in B light also led to a strong increase (~290%) in cotyledon opening angle compared with *cry1* mutant seedlings (Fig. S7 at Dryad). These results suggest that MKK10-MPK6 acts downstream of phyB in regulating cotyledon opening and that phyA or CRY1 share partially overlapping functions with phyB (Casal and Boccalandro, 1995; Casal and Mazzella, 1998).

### PIF3 regulates MKK10-MPK6-mediated cotyledon opening

**Fig. 8.**
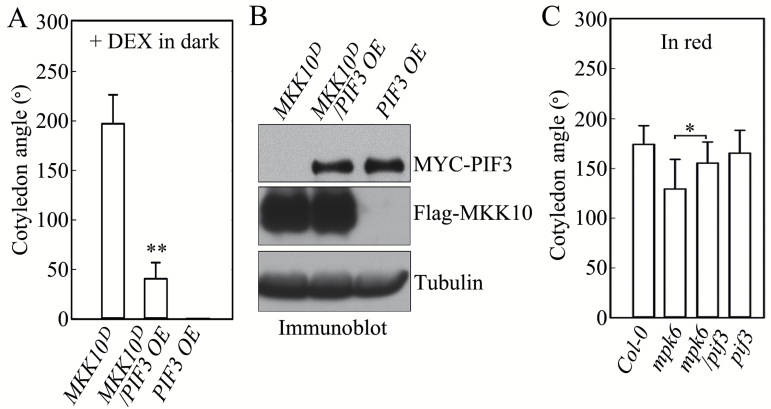
PIF3 functions downstream of MKK10-MPK6 in regulating cotyledon opening in seedlings in R light. (A) Cotyledon opening angles of *MKK10*^*D*^, *MKK10*^*D*^*/PIF3 OE*, and *PIF3 OE* seedlings in the dark. Data are means±SD (*n*=30). Asterisks indicate significant differences between *MKK10*^*D*^ and *MKK10*^*D*^*/PIF3 OE*. (B) Protein levels of MKK10^D^ and MYC-PIF3 detected by immunoblot analysis with anti-Flag and anti-MYC antibodies. (C) Cotyledon opening angles of Col-0, *mpk6*, *pif3*, and *mpk6/pif3* seedlings in R light. Data are means±SD (*n*=30). Asterisks indicate significant differences between *mpk6* and *mpk6/pif3* seedlings. (Student’s *t*-test, *P<0.05, **P<0.01).

PIF3, a repressor of photomorphogenesis, plays a pivotal role in phyB-mediated R light responses (Leivar and Monte, 2014; Leivar and Quail, 2011). We investigated whether PIF3 is involved in MKK10-MPK6-regulated cotyledon opening by generating and analyzing *MKK10*^*D*^/*PIF3 OE* and *mpk6*/*pif3* lines (Fig. S6 at Dryad). As shown in Fig. 8A, the MKK10^D^-induced cotyledon opening phenotype in the dark was repressed when *MYC*-*PIF3* was overexpressed in *MKK10*^*D*^/*PIF3 OE* seedlings. The increased cotyledon opening angle caused by the induction of MKK10^D^ was 79% smaller in *MKK10*^*D*^/*PIF3 OE* seedlings in the dark than in *MKK10*^*D*^ seedlings. Immunoblot analysis revealed comparable levels of MKK10^D^ in *MKK10*^*D*^ and *MKK10*^*D*^/*PIF3 OE* seedlings, and MYC-PIF3 in *MKK10*^*D*^/*PIF3 OE* and *PIF3 OE* seedlings (Fig. 8B). The reduced cotyledon opening angle caused by the loss of function of MPK6 was partially rescued in *mpk6*/*pif3* seedlings in R light (Fig. 8C). These results suggest that PIF3 mediates the cotyledon opening process regulated by the MKK10-MPK6 cascade. However, overexpression of PIF3 did not completely abolish the MKK10^D^-induced cotyledon opening phenotype, implying that additional factors other than PIF3 (e.g. other PIFs) are also involved in this process.

### MKK10-MPK6 induces PIF3 phosphorylation and accelerates PIF3 degradation

The phosphorylation and subsequent degradation of PIF3 are required for the promotion of photomorphogenesis when seedlings are exposed to light (Leivar and Monte, 2014; Leivar and Quail, 2011); however, little is known about the kinase(s) that phosphorylate PIF3. Since PIF3 is involved in MKK10-MPK6-regulated cotyledon opening, we investigated whether MKK10-MPK6 phosphorylates PIF3. The interaction between MPK6 and PIF3 and the phosphorylation of PIF3 by MKK10-MPK6 were examined.

**Fig. 9.**
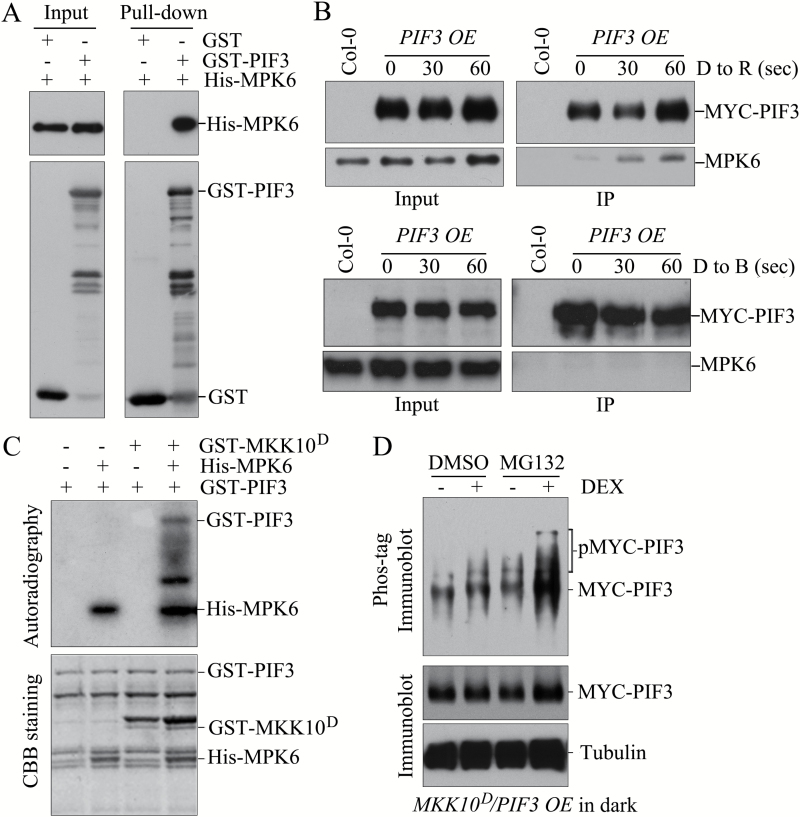
MPK6 phosphorylates PIF3 and accelerates its degradation. (A) GST pull-down assay. GST or GST-PIF3 was bound to Glutathione Sepharose 4B beads and then incubated with His-MPK6. Proteins bound to beads were analyzed by immunoblot analysis with anti-His antibody (upper panel) and anti-GST antibody (lower panel). (B) Co-immunoprecipitation of MPK6 and MYC-PIF3. Dark-grown *PIF3 OE* seedlings were exposed to 30 μmol m^–2^ s^–1^ R light or 8 μmol m^–2^ s^–1^ B light for 30 and 60 s. Proteins were extracted from the seedlings, and an immunoprecipitation (IP) assay was performed using anti-MYC beads. Proteins bound to beads were analyzed by immunoblot analysis with anti-MYC antibody (upper panels) and anti-MPK6 antibody (lower panels). (C) Phosphorylation of PIF3 by MKK10-MPK6 *in vitro*. His-MPK6 was activated by GST-MKK10^D^ and used to phosphorylate GST-PIF3. After the reaction, the proteins were separated by SDS-PAGE and visualized by Coomassie Brilliant Blue (CBB) staining (lower panel). The phosphorylated proteins were detected by autoradiography (upper panel). (D). Phosphorylation of PIF3 by MKK10-MPK6 *in vivo*. *MKK10*^*D*^/*PIF3 OE* seedlings were grown on medium with or without DEX in the dark and then treated with MG132 (50 μM final concentration). Protein extracts from seedlings were separated on an SDS-PAGE gel with Phos-tag reagent and transferred to a nitrocellulose membrane. MYC-PIF3 was detected by immunoblot analysis with anti-MYC antibody (upper panel). A standard immunoblot assay was performed to detect total MYC-PIF3 protein in the extracts.

We performed pull-down experiments using His-MPK6 with GST-PIF3 or GST as input proteins to detect the interaction between MPK6 and PIF3 *in vitro*. As shown in Fig. 9A, anti-GST antibody detected both GST and GST-PIF3, and anti-His antibody detected His-MPK6 in both input samples, indicating the successful loading of GST, GST-PIF3, and His-MPK6. After incubation and pull-down with GST-affinity beads, we found that anti-His antibody detected His-MPK6 in the GST-PIF3 pull-down sample but not in the GST pull-down sample. The His-MPK6 was pulled down by GST-PIF3, suggesting that MPK6 interacted with PIF3 *in vitro*. We performed Co-IP experiments with MPK6 and PIF3 using *PIF3 OE* seedlings that were grown in the dark and transferred to the R light condition for 0, 30, and 60 s to test the interaction of PIF3 with MPK6 *in vivo* and the regulation of this interaction by R light. As shown in Fig. 9B, a lower level of MPK6 co-immunoprecipitated with MYC-PIF3 in the samples from seedlings before transfer to R light (0 s); however, after the seedlings were exposed to R light for 30 or 60 s, the levels of MPK6 that co-immunoprecipitated with MYC-PIF3 increased significantly. Co-IP of PIF3 with MPK6 suggested that MPK6 interacted with PIF3 *in vivo*. Exposure of seedlings to B light did not enhance the interaction of PIF3 with MPK6. To confirm the interaction of these proteins, we investigated the transient co-expression of MPK6-mCherry and PIF3-GFP in tobacco leaves. MPK6 and PIF3 co-localized in nuclear bodies (Fig. S8 at Dryad). Our findings that R light treatment increased the interaction of MPK6 with PIF3 and that MPK6 was activated by R light suggest that activation of MPK6 strengthens its interaction with PIF3.

We performed phosphorylation experiments via *in vitro* kinase activity assays using GST-MKK10^D^, His-MPK6, and GST-PIF3. As shown in Fig. 9C, GST-PIF3 was phosphorylated in the presence of both GST-MKK10^D^ and His-MPK6 in the reaction, whereas GST-PIF3 was not phosphorylated in the presence of GST-MKK10^D^ or His-MPK6 alone. These results suggested that activation of MPK6 by MKK10 phosphorylated PIF3 *in vitro*. We also examined the phosphorylation of MYC-PIF3 in *MKK10*^*D*^/*PIF3 OE* seedlings grown in the dark before or after MKK10^D^ induction using Phos-tag mobility shift assays. Because the phosphorylation of PIFs is degraded through the 26S proteasome-mediated pathway (Al-Sady *et al.*, 2006; Ni *et al.*, 2013; Park *et al.*, 2004; Shen *et al.*, 2005; Shen *et al.*, 2007), we also used MG132, a 26S proteasome inhibitor, in this experiment. As shown in Fig. 9D, in the absence of MG132, the strengths and patterns of the MYC-PIF3 bands in seedlings before and after MKK10^D^ induction (DMSO with or without DEX) did not significantly differ. However, when seedlings were pretreated with MG132, multiple newly emerged bands were observed and the strength of the formerly existed bands of MYC-PIF3 in the sample of seedlings after MKK10^D^ induction (MG132 with DEX) increased. These results demonstrate that MYC-PIF3 is phosphorylated and that the phosphorylation of PIF3 by MKK10-MPK6 accelerates its degradation *in vivo*.

### Activation of MKK10-MPK6 regulates transcription of light-responsive genes

**Fig. 10.**
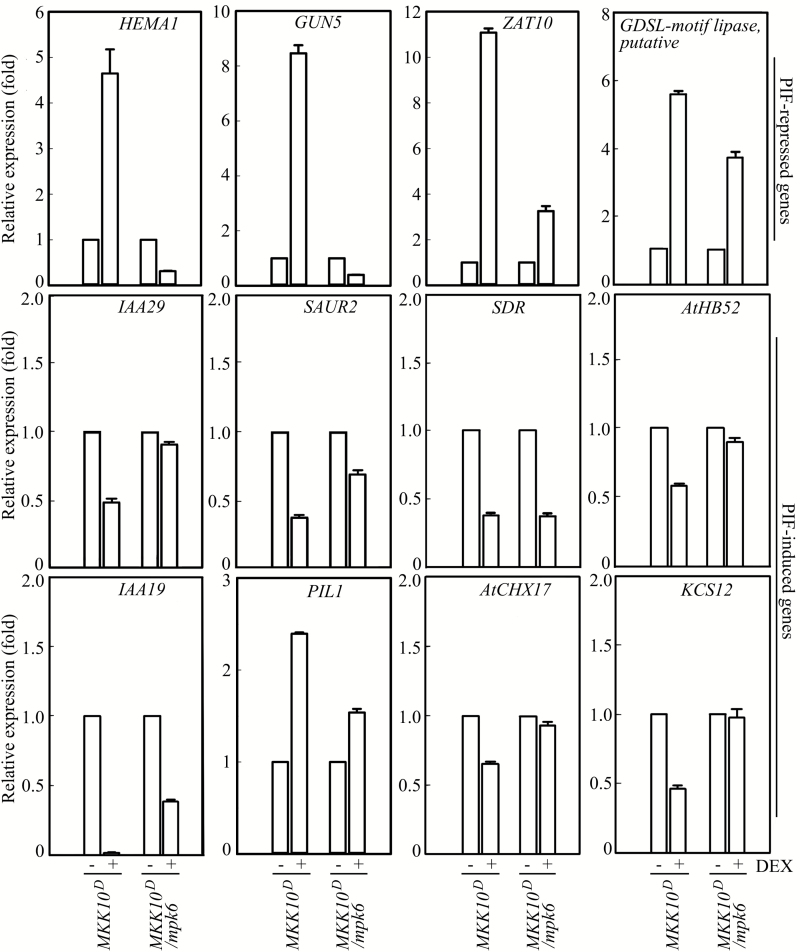
Q-PCR detection of PIF-induced or PIF-repressed gene transcription in *MKK10*^*D*^ and *MKK10*^*D*^*/mpk6* seedlings. Seedlings were grown on medium with or without DEX in the dark for 4 days. The transcription levels of the indicated genes were monitored by Q-PCR. Data are means±SD of three biological replicates.

The transcription of many genes is regulated in seedlings during photomorphogenesis (Jiao *et al.*, 2007; Quail, 2007). PIFs such as PIF1, PIF3, PIF4, and PIF5 play a central role in regulating the transcription of these genes (Leivar *et al.*, 2009; Zhang *et al.*, 2013). Because MKK10-MPK6 phosphorylated PIF3 and accelerated its degradation (Fig. 9), we speculated that the transcription of some PIF-regulated genes would be altered after MKK10-MPK6 activation. We performed Q-PCR to measure the transcript levels of several genes that are induced or repressed by PIFs, as shown previously, in *MKK10*^*D*^ and *MKK10*^*D*^*/mpk6* seedlings with or without MKK10^D^ induction. As shown in Fig. 10, the transcription of some PIF-repressed genes, e.g. *HEMA1*, *GUN5*, *ZAT10*, and a gene encoding a protein with a putative GDSL motif (*GDSL-motif lipase* putative), was strongly induced in *MKK10*^*D*^ seedlings after MKK10^D^ induction, whereas the induction levels of *ZAT10* and *GDSL-motif lipase* putative were partially compromised and *HEMA1* and *GUN5* transcript levels were reduced in *MKK10*^*D*^*/mpk6* seedlings. The transcription of PIF-induced genes, including *IAA29*, *SAUR2*, *SDR*, *AtHB52*, *IAA19*, *AtCHX17*, and *KCS12*, was reduced in *MKK10*^*D*^ seedlings after MKK10^D^ induction, whereas the reduced transcription of these genes (except for *SDR*) was partially or fully reversed in *MKK10*^*D*^*/mpk6* seedlings. However, the transcription of some genes, e.g. *PIL1*, which is induced by PIFs (Luo *et al.*, 2014; Zhang *et al.*, 2013), was induced in *MKK10*^*D*^ seedlings and reduced in *MKK10*^*D*^*/mpk6* seedlings. These results suggest that the activation of MKK10-MPK6 regulates the transcription of some PIF-regulated genes.

## Discussion

Genetic screening and molecular studies have revealed that many genes are involved in the regulation of seedling photomorphogenesis. The mutation of photoreceptor genes suppresses seedling photomorphogenesis under specific spectral compositions of light, leading to the following phenotypes: *phyA* seedlings show elongated hypocotyls in FR and B light and exaggerated cotyledons in FR light; *phyB* seedlings display elongated hypocotyls and reduced cotyledon opening in R and white light; *cry1* seedlings show elongated hypocotyls and reduced cotyledon opening in B and white light; *phyA/phyB* double mutant seedlings exhibit increased hypocotyl elongation in FR, R, B, and white light, exaggerated cotyledons in FR and R light, and reduced cotyledon opening in white light; *phyA/phyB/cry1* triple mutant seedlings show severe cotyledon-opening and hypocotyl-elongation phenotypes (Arsovski *et al.*, 2012; Li *et al.*, 2011; Mockler *et al.*, 1999; Neff and Chory, 1998; Reed *et al.*, 1994; Yu *et al.*, 2010). Mutation analysis showed that some transcription factors act downstream of photoreceptors as either positive or negative regulators of seedling photomorphogenesis. For example, *HY5* (a bZIP transcription factor) and *HFR1* (an atypical bHLH transcription factor) are positive regulators of this process. Whereas *hy5* mutant seedlings show elongated hypocotyls in the light, seedlings overexpressing truncated HY5 (COP1-interactive domain deleted) exhibit shortened hypocotyls (Ang *et al.*, 1998; Ang and Deng, 1994; Arsovski *et al.*, 2012; Lee *et al.*, 2007; Li *et al.*, 2011; Oyama *et al.*, 1997; Ulm *et al.*, 2004). Moreover, *hfr1* mutant seedlings have elongated hypocotyls and reduced cotyledon opening in FR and B light (Duek and Fankhauser, 2003; Fairchild *et al.*, 2000; Fankhauser and Chory, 2000; Soh *et al.*, 2000), whereas seedlings overexpressing truncated HFR1 (N-terminal 105 amino acids deleted) have shortened hypocotyls in the dark and FR light and open cotyledons in the dark (Yang *et al.*, 2003). A small subset of PIFs, including PIF1, PIF3, PIF4 and PIF5, are key negative regulators of photomorphogenesis (Leivar and Monte, 2014; Leivar and Quail, 2011; Monte *et al.*, 2007; Seluzicki *et al.*, 2017; Zhang *et al.*, 2013). In the dark, quadruple mutant (*pifq*) seedlings with these four PIF genes mutated exhibit a partially constitutive photomorphogenic phenotype, that is, shortened hypocotyls and open cotyledons (Leivar *et al.*, 2008). However, the signaling modules that link photoreceptors and these transcription factors are far from clear.

MAPK cascades are well-known signaling modules that perceive signals from receptors/sensors and transduce these signals to elicit cellular responses (Andreasson and Ellis, 2010; Colcombet and Hirt, 2008; Meng and Zhang, 2013). In this study, we explored whether MAPK cascades are involved in regulating the responses of *Arabidopsis* seedlings to R light and, if so, which MAPK cascades are involved, and how. We used cotyledon opening angle in seedlings as the primary parameter for phenotypic observations. The strong activation of MPK6 by R light and the reduced cotyledon opening in *mpk6* mutant seedlings compared with Col-0 seedlings under R light suggested that MPK6 activity is involved in R-light-regulated cotyledon opening in seedlings. To demonstrate the sufficiency of MPK6 activity for the regulation of cotyledon opening, seedlings with long-lasting MPK6 activation must be examined in the dark. The active forms of MKK1 to MKK5, MKK7, and MKK9 have previously been reported to activate MPK6, with the activation of MPK6 by different MKKs in plants affecting distinct biological functions (Jia *et al.*, 2016; Ren *et al.*, 2002; Sethi *et al.*, 2014; Takahashi *et al.*, 2007; Teige *et al.*, 2004; Xing *et al.*, 2008; Xu *et al.*, 2008); however, none of these MKKs has been implicated in the activation of MPK6 to regulate cotyledon opening. We therefore generated transgenic plants overexpressing active MKKs (MKK1^DD^ to MKK9^DD^ and MKK10^D^) and observed cotyledon opening in seedlings in the dark. The overexpression of *MKK1*^*DD*^ to *MKK8*^*DD*^ did not induce cotyledon opening, and the overexpression of *MKK9*^*DD*^ only weakly induced cotyledon opening in the dark; however, surprisingly, the overexpression of *MKK10*^*D*^ strongly induced cotyledon opening in the dark. Because the biological function of MKK10 has not previously been reported, our results provide the first evidence that MKK10 is the primary MKK that regulates cotyledon opening in seedlings.

*GUS* driven by the *MKK10* promoter was specifically expressed in cotyledon, further supporting the role of MKK10 in cotyledon opening (Fig. S3 at Dryad). The activation of MPK6 by MKK10 *in vitro* and *in vivo*, and the abolishment of the *MKK10*^*D*^-induced cotyledon opening phenotype in *MKK10*^*D*^/*mpk6* seedlings, demonstrated that MKK10 indeed functions upstream of MPK6 in R-light-regulated cotyledon opening in seedlings. Considering the function of MKK10-MPK6 in R-light-regulated cotyledon opening in seedlings and the predominant role of phyB in R light perception, we reasoned that the MKK10-MPK6 cascade might function downstream of phyB. This speculation was further supported by the findings that R-light-induced MPK6 activation was significantly reduced in *phyB* mutant seedlings and that overexpression of *MKK10*^*D*^ rescued the suppressed cotyledon opening in the *phyB* mutant background. Overexpression of *MKK10*^*D*^ increased the cotyledon opening angle in the *cry1* or *phyA* mutant background, whereas the *mpk6* and *mkk10* mutants did not show reduced cotyledon opening angles in FR or B light, suggesting that phyA and CRY1 play a minor role in the MKK10-MPK6-mediated cotyledon opening signaling pathway. The conditional synergistic interaction between phyB and CRY1 or phyA in have been reported previously (Casal and Boccalandro, 1995; Casal and Mazzella, 1998).

Phosphorylation of target proteins is a primary mechanism by which MAPK cascades transduce signals. The phosphorylation and subsequent degradation of PIFs are required for seedling photomorphogenesis (Bu *et al.*, 2011; Lorrain *et al.*, 2008; Ni *et al.*, 2013; Shen *et al.*, 2008; Shen *et al.*, 2007). Multiple phosphorylation residues have been identified in PIF proteins; for example, over 20 phosphorylation sites are present in PIF3, a founding member of the PIF subset (Bu *et al.*, 2011; Ni *et al.*, 2013). Multiple light-regulated kinases are likely responsible for the phosphorylation of PIF3 (Xu *et al.*, 2015); however, to date, the only kinases that have been shown to phosphorylate PIF3 are *Avena sativa* AsphyA and Arabidopsis BIN2 and PPKs (Ling *et al.*, 2017; Shin *et al.*, 2016). Our results indicate that MPK6 is another kinase responsible for the phosphorylation and functional regulation of PIF3: MPK6 was activated by R light; MPK6 interacted with PIF3 *in vitro* and *in vivo*, and the interaction in seedlings was enhanced by R light; the activation of MPK6 by MKK10 resulted in PIF3 phosphorylation and accelerated its degradation; MKK10-induced cotyledon opening in *MKK10*^*D*^ seedlings was greatly suppressed by the overexpression of PIF3; and the MKK10-MPK6 cascade regulates the transcription of several PIF3-regulated genes. These findings suggest that MKK10-MPK6-PIF3 is a module that functions downstream of phyB to regulate cotyledon opening in R light. PIF3 has been linked to ethylene signaling (Zhong *et al.*, 2014). However, ethylene signaling was not conclusively shown to be involved in MKK10-MPK6-PIF3-regulated cotyledon opening in this study, as the MKK10 variant with the cotyledon opening phenotype (*MKK10*^*D*^) only showed lower levels of induction of *ERFs* (less than 2-fold for all four *ERFs*) (Fig. S9 at Dryad).

Inhibited hypocotyl elongation was also observed in seedlings overexpressing *MKK10*^*D*^ and in lines crossed with these plants. However, we believe that this phenotype does not reflect a true biological function of MKK10 for two reasons: first, *MKK10* was not expressed in hypocotyls, as revealed by examining the expression of *GUS* driven by the native promoter of *MKK10*; second, no obvious hypocotyl phenotype was detected in *mpk6* or *mkk10* seedlings.

**Fig. 11.**
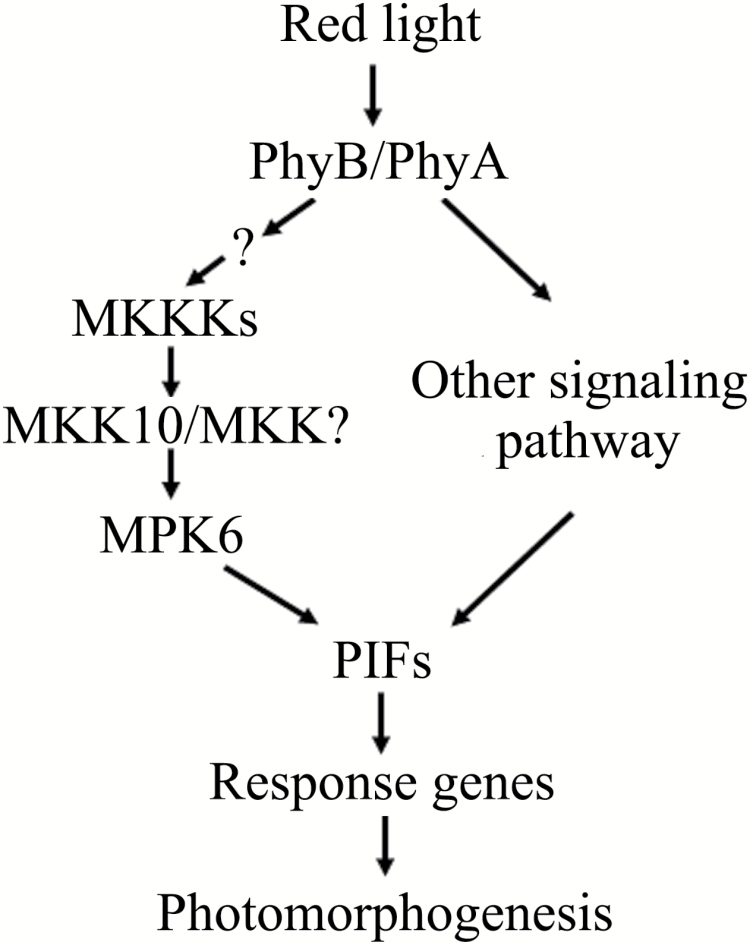
Proposed working model for MKK10-MPK6 cascade-mediated regulation of cotyledon opening in seedlings.

Based on the current and previous results, we propose a working model for the role of a MAPK cascade in R-light-regulated cotyledon opening (Fig. 11). When seedlings are exposed to R light, phyB perceives the light signal and is activated; active phyB in turn activates a currently unidentified MAPKKK (labeled ‘MKKKs’ in Fig. 11) directly or through an additional mediator, and the unidentified MKKK(s)-MKK10-MPK6 cascade is sequentially phosphorylated and activated; the active MPK6 phosphorylates PIF3 and accelerates its degradation; and PIF3-repressed or -induced genes are inversely regulated to promote cotyledon opening in seedlings. Perhaps the phosphorylation and degradation of other PIFs or transcription factors that are functionally redundant with PIF3 are also involved in this process. Identifying the unknown MKKK(s) and exploring the MPK6 phosphorylation sites in PIF3 and the effect of MPK6 phosphorylation on PIF3 function in future studies will help uncover the mechanisms by which the MKK10-MPK6 cascade regulates cotyledon opening in R light.

## Data deposition

The following tables and figures are available at Dryad Data Repository: http://dx.doi.org/10.5061/dryad.hq7b8.

Table S1. Oligonucleotides used in this study.

Table S2. Accession numbers of genes used in this study.

Fig. S1. Hypocotyl length of Col-0, *mpk3*, and *mpk6* seedlings under different light conditions.

Fig. S2. Sequence alignment of Arabidopsis MKKs.

Fig. S3. Kinase activity assay of *MKK10*^*D*^ seedlings.

Fig. S4. *MKK10* gene expression pattern in seedlings.

Fig. S5. The CRISPR construct used to create the *mkk10* mutant.

Fig. S6. *pif3* mutant identification.

Fig. S7. Analysis of cotyledon opening in *MKK10*^*D*^*/cry1* mutant seedling.

Fig. S8. Co-localization analysis of MPK6 and PIF3.

Fig. S9. Q-PCR detection of *ERF* gene transcription in transgenic seedlings harboring MKK10 variants.

Supplementary Table S1-S2 and Figure S1-S9
