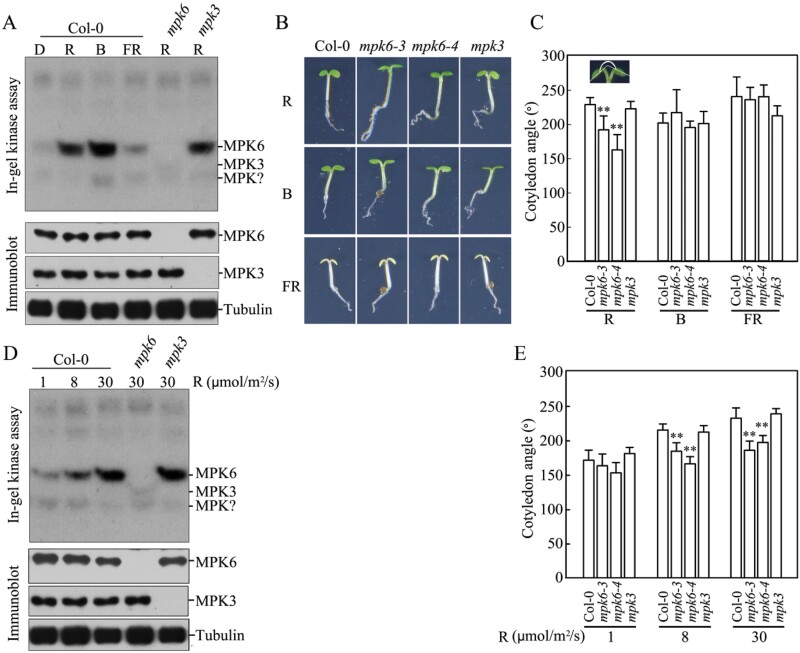# Correction to: Arabidopsis MKK10-MPK6 mediates red-light-regulated opening of seedling cotyledons through phosphorylation of PIF3

**DOI:** 10.1093/jxb/erac310

**Published:** 2022-09-14

This is a correction to: Xiaoyun Xin, Wenhao Chen, Bo Wang, Fan Zhu, Yuan Li, Hailian Yang, Jigang Li, Dongtao Ren, Arabidopsis MKK10-MPK6 mediates red-light-regulated opening of seedling cotyledons through phosphorylation of PIF3, *Journal of Experimental Botany*, Volume 69, Issue 3, 23 January 2018, Pages 423–439, https://doi.org/10.1093/jxb/erx418

In Figure 1D of the original version of this article the immunoblot image for MPK6 was cropped incorrectly to include a superfluous lane containing a loading control. A corrected version of the figure is included below. The authors would like to apologise for this error.

These details have been corrected only in this correction notice to preserve the published version of record.