# Supplementary material for: Arabidopsis MKK10-MPK6 mediates red-light-regulated opening of seedling cotyledons through phosphorylation of PIF3
Source: J Exp Bot. 2017 Dec 13;69(3):423–39. doi: 10.1093/jxb/erx418 (PMC5853512; doi:10.1093/jxb/erx418)
Supplement: Supplementary Table S1-S2 and Figure S1-S9 [file erx418_suppl_supplementary_table_s1_s2_and_figure_s1_s9.pdf]

Supplementary data

Supplementary Table S1. Oligonucleotides used in this study

Oligo name	Oligonucleotide (5'-3')
	Primers for genomic screen of mutants
mpk6-3 (Salk_127507)	
mpk6-3-LP	CTCTGGCTCATCGCTTATGTC
mpk6-3-RP	ATCTATGTTGGCGTTTGCAAC
mpk6-4 (Salk_062471)	
mpk6-4-LP	GTCCAGGGAAGAGTGGCTTAC
mpk6-4-RP	GCAGTTCGGCTATGAATTCTG
pif3 (Salk_030753)	
pif3-1-LP	AGTCTGTTGCTTCTGCTACGC
pif3-1-RP	AAGAACCGGCAAAGATACCAC
mkk9 (SAIL_60_H06)	
mkk9-LP	ACTAGTATGGCTTTAGTACGTGAAC
mkk9-RP	CCCGGGAAGATCTTCC
T-DNA	
LBa1	TGGTTCCGTAGTGGGCCATCG
LB3	TAGCATCTGAATTTCATAACCAATCTCGATACAC
	Primers for RT-PCR screen of mutants
MPK6 (At2g43790)	
MPK6 RT-F	ATGGACGGTGGTTCAGGTCA
MPK6 RT-R	CTATTGCTGATATTCTGGAT
PIF3 (At1g09530)	
PIF3 RT-F	GGACTCATGTAGAAAAGACC
PIF3 RT-R	CAGAGACACGTGGAATACCC
	Primers for vector constructs
MKK10 (At1g32320)	
MKK10-Nde-F	GGGTTTCATATGACACTTGTTAGAGAACGAC
Spe Sal-MKK10-R	ACTAGTGTCGACCTATCTGTTTTTCACAAAAG
MKK10-K77R-F	AAAACGCTTTACGCCTTAAGAGTCCTCCGGCCAAATCTC
MKK10-K77R-R	GAGATTTGGCCGGAGGACTCTTAAGGCGTAAAGCGTTTT
MKK10-S197D-F	GCCGGAGGAGACTATGGAGATAATGGGACATGTGCTTAT
MKK10-S197D-R	ATAAGCACATGTCCCATTATCTCCATAGTCTCCTCCGGC
MKK10Pro-F	AACCGAAATCTCTCTGCGCCC
MKK10Pro-R	CTAGGTTATTTGCTTGCACTTGTGG
DT1-BsF	ATATATGGTCTCGATTGTTGAGCAAGTAACGCGTCAGTT

DT1-F0	TGTTGAGCAAGTAACGCGTCA GTTTTAGAGCTAGAAATAGC
DT2-R0	AACTAAACGATCTCGAGAAACTCAATCTCTTAGTCGACTCTAC
DT2-BsR	ATTATTGGTCTCGAAACTAAACGATCTCGAGAAACTC
U626-IDF	TGTCCCAGGATTAGAATGATTAGGC
U629-IDF	TTAATCCAAACTACTGCAGCCTGAC
U629-IDR	AGCCCTCTTCTTTCGATCCATCAAC
PIF3 (At1g09530)	
Nde-PIF3-F	CATATGATGCCTCTGTTTGAGCTTTT
Spe Sal-PIF3-R	ACTAGTGTCGACTCACGACGATCCACAAAACTGATC
PIF3-KpnI-R	GGTACCCGACGATCCACAAAACTGATCAGA
MPK6 (At2g43790)	
MPK6-SpeI-F	ACTAGTATGGACGGTGGTTCAGGTCAACCG
MPK6-KpnI-BglII-R	AGATCTGGTACCTTGCTGATATTCTGGATTGA
mCherry	
mCherry-KpnI-F	GGTACCATGGTGAGCAAGGGCGAGGAG
mCherry-SacI-R	GAGCTCCTACTTGTACAGCTCGTCCATG
	Primers for Q-PCR
UBQ10 (At4g05320)	
UBQ10-QF	GATCTTTGCCGGAAAACAATTGGAGGATGGT
UBQ10-QR	CGACTTGTCATTAGAAAGAAAGAGATAACAGG
PIL1 (At2g46970)	
PIL1-QF	AAATTGCTCTCAGCCATTCGTGG
PIL1-QR	TTCTAAGTTTGAGGCGGACGCAG
GUN5 (At5g13630)	
GUN5-QF	GAGCTTGATGGAGCGATGGAGCCAATCGTTTTC
GUN5-QR	GTCCCTACATTACCTTTATCAGGTGGGAAACTG
HEMA1 (At1g58290)	
HEMA1-QF	ACCAGACGAGGTTGGATCCAAAAAGCTCGTTGT
HEMA1-QF	ATAGCAAGCTTCTCACGCATCTCAACAGGAGCT
ZAT10 (At1g27730)	
ZAT10-QF	ATCACACGTTTGCACCATCT
ZAT10-QR	TGCTAACGTGGCTAGTGGAC
IAA29 (At4g32280)	
IAA29-QF	CACCATCATTGCCCGTATCA
IAA29-QR	CCACAGTAGCCGTTGTTGGA
SAUR2 (At4g13790)	
SAUR2-QF	CGGAATCATTATCAACGCCTAAA
SAUR2-QR	TGTTCAAGTAACAAACCGGAACA

SDR (At5g02540)	
SDR-QF	ATGAGCTCTCCCGTCAGCTTCAGG
SDR-QR	CTCCCTTCACACTTGGATGCAGAGC
IAA19 (At3g15540)	
IAA19-QF	TTCCGTGGCATCGGTGTGGC
IAA19-QR	GCTGCAGCCCAAACCCGGTAG
ATHB52 (At5g53980)	
ATHB52-QF	TGGTTCCAAAACAAGCGAGCCAGG
ATHB52-QR	GTCGGAGAGAGCTGCTTCGTGC
ATCHX17 (At4g23700)	
AtCHX17-QF	CGCTTAAGAGATTTGGGATCGAGCTC
AtCHX17-QR	GCGTTGCCTCTTCTCATACTCCAGATC
GDSL (At1g28570)	
GDSL-QF	GAAGAGATAGTTTCAATAACAAGCGG
GDSL-QR	GCCGTACAGCTTAGTAGCTTCAAGG
KCS12 (At2g28630)	
KCS12-QF	TACAAGATGAGAGACGACGTCAAAGTC
KCS12-QR	CACATCCACCGGACCGGAACAAACAG
ERF1(At3g23240)	
ERF-QF	CGGCGGAGAGAGTTCAAGAGTC
ERF-QR	TCCCACTATTTTCAGAAGACCCC
ERF2(At5g47220)	
ERF-QF	GGAGGTTTGCCATTGAAAGA
ERF-QR	CAGCTCAAGTCCGATGATGA
ERF5(At5g47230)	
ERF-QF	GTCTCAAATTCGTAAACCGCCA
ERF-QR	CCCCACGGTCTTTGTCTTACTC
ERF6(At4g17490)	
ERF-QF	GGAGAAGAGGCATTACAGAGGAGTG
ERF-QR	TCGTAGTCTAAACGCTTCTTTGTCG

Supplementary Table S2. Accession number of genes used in this article.

Gene	Accession number	Gene	Accession number	Gene	Accession number
MKK1	At4g26070	*MPK10*	At3g59790	*PIL1*	At2g46970
MKK2	At4g29810	*MPK12*	At2g46070	*SAUR2*	At4g13790
MKK3	At5g40440	*PIF3*	At1g09530	*AtCHX17*	At4g23700
MKK4	At1g51660	*phyA*	At1g09570	*KCS12*	At2g28630
MKK5	At3g21220	*phyB*	At2g18790	*ERF1*	At3g23240
MKK6	At5g56580	*HEMA1*	At1g58290	*ERF2*	At5g47220
MKK7	At1g18350	*GUN5*	At5g13630	*ERF5*	At5g47230
MKK8	At3g06230	*ZAT10*	At1g27730	*ERF6*	At4g17490
MKK9	At1g73500	*IAA29*	At4g32280	*UBQ*	At4g05320
MKK10	At1g32320	*SDR*	At5g02540	*GDSL-moti[illegible]*	
MPK3	At3g45640	*AtHB52*	At5g53980	*lipase*	At1g28570
MPK6	At2g43790	*IAA19*	At3g15540	*putative*	

Note: Sequence data for this article can be found in Arabidopsis Genome initiative database under the following accession numbers.

Fig S1

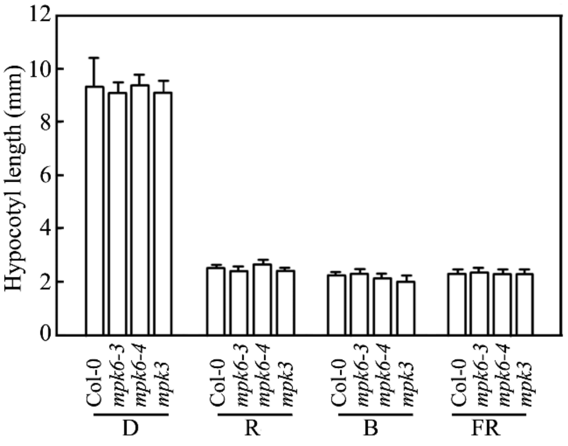

Fig. S2

A

```
                        D                       D
                        ↓                       ↓
MKK1    S K I L T S T S S L A N S F V G T Y P Y   231
MKK2    S T V M T N T A G L A N T F V G T Y N Y   242
MKK3    S A G L E N S M A M C A T F V G T V T Y   248
MKK4    S R I L A Q T M D P C N S S V G T I A Y   237
MKK5    S R I L A Q T M D P C N S S V G T I A Y   228
MKK6    S A S L A S S M G Q R D T F V G T Y N Y   234
MKK7    S K I I T R S L D Y C N S Y V G T C A Y   206
MKK8    S K I V V R S L N K C N S F V G T F A Y   208
MKK9    S K I L V R S L D S C N S Y V G T C A Y   208
MKK10   S R I V A G - G D Y G - S N - G T C A Y   203
```

B

```
                            R
                            ↓
MKK9    H K T T S E I Y A L K T V N G D M D P I F T   87
MKK10   H R R T K T L Y A L K V L R P N - - - - L N   84
```

Fig. S3

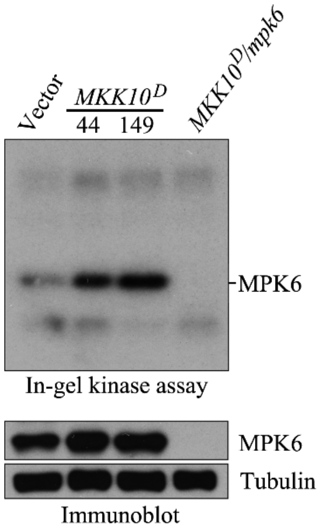
Vector
MKK10^D
44
149
MKK10^D/mpk6
MPK6
In-gel kinase assay
MPK6
Tubulin
Immunoblot

Fig. S4

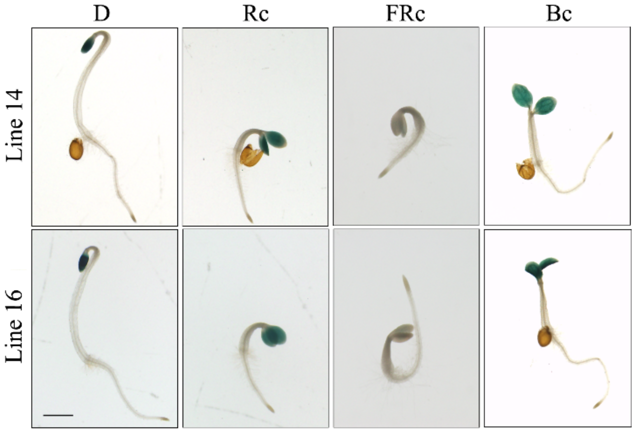

Fig. S5

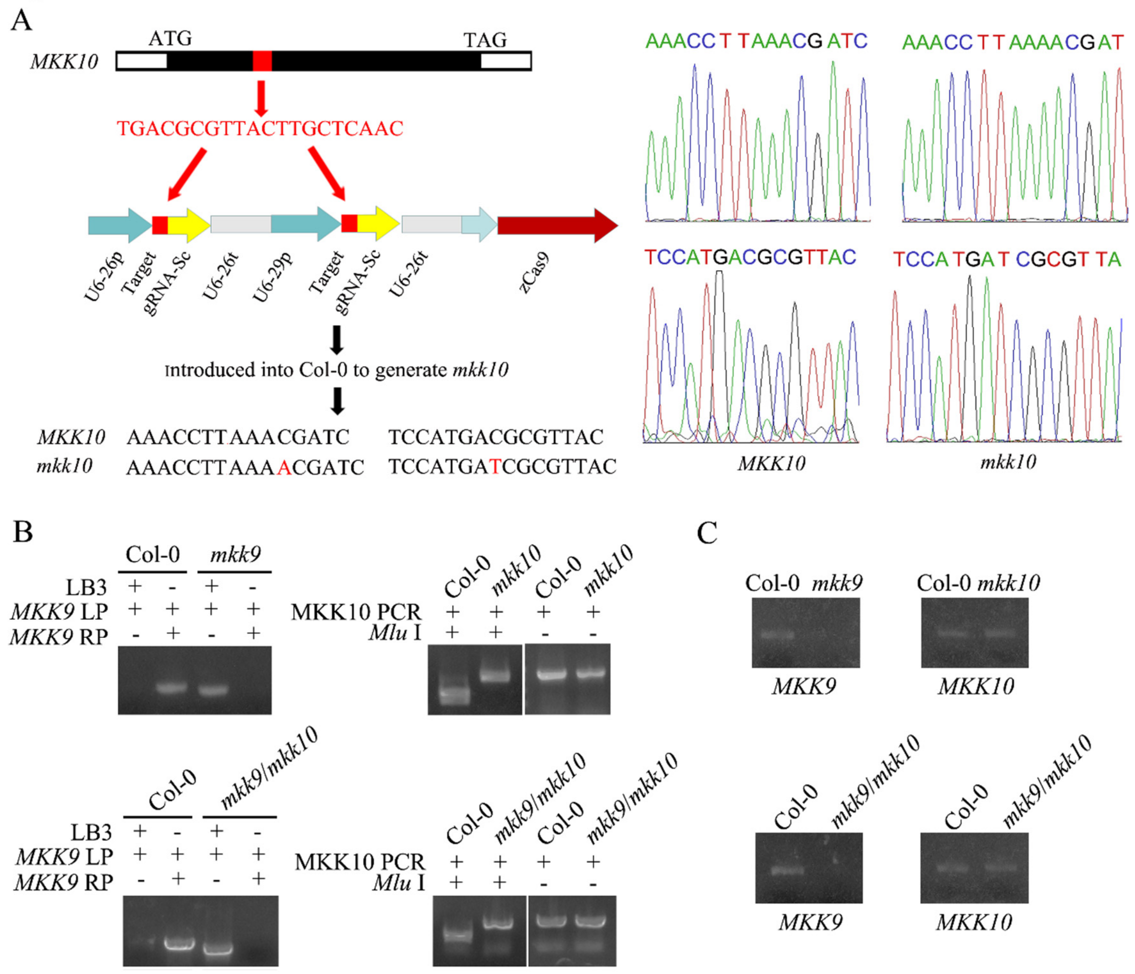

A
MKK10
ATG
TAG
TGACGCGTTACTTGCTCAAC
U6-26p
Target
gRNA-Sc
U6-26t
U6-29p
Target
gRNA-Sc
U6-26t
zCas9
Introduced into Col-0 to generate mkk10
MKK10 AAACCTTAAACGATC TCCATGACGCGTTAC
mkk10 AAACCTTAAAACGATC TCCATGATCGCGTTAC
AAACCTTAAACGATC
AAACCTTAAAACGAT
TCCATGACGCGTTAC
TCCATGATCGCGTTA
MKK10
mkk10
B
Col-0
mkk9
LB3
MKK9 LP
MKK9 RP
MKK10 PCR
Mlu I
Col-0
mkk10
mkk9/mkk10
C
Col-0 mkk9
Col-0 mkk10
MKK9
MKK10
Col-0
mkk9/mkk10

Fig. S6

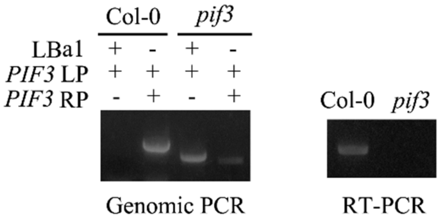

Fig.S7

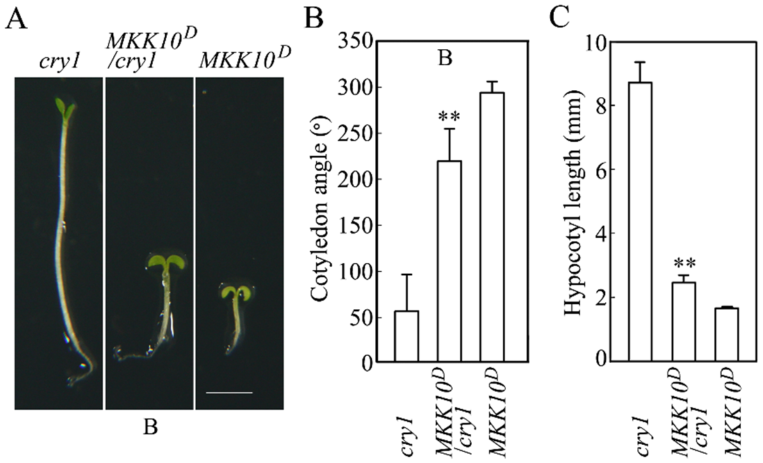
A
cry1
MKK10^D /cry1
MKK10^D
B
B
350
300
250
200
150
100
50
0
Cotyledon angle (°)
**
cry1
MKK10^D /cry1
MKK10^D
C
10
8
6
4
2
0
Hypocotyl length (mm)
**
cry1
MKK10^D /cry1
MKK10^D

Fig.S8

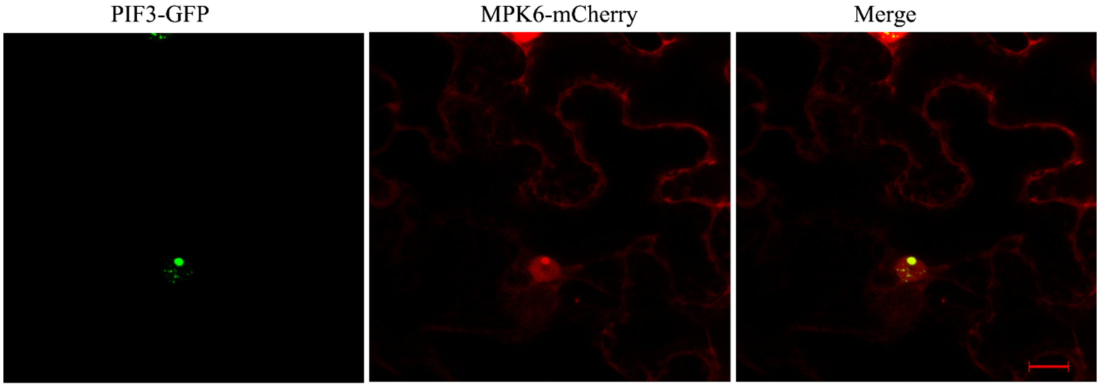

Fig. S9

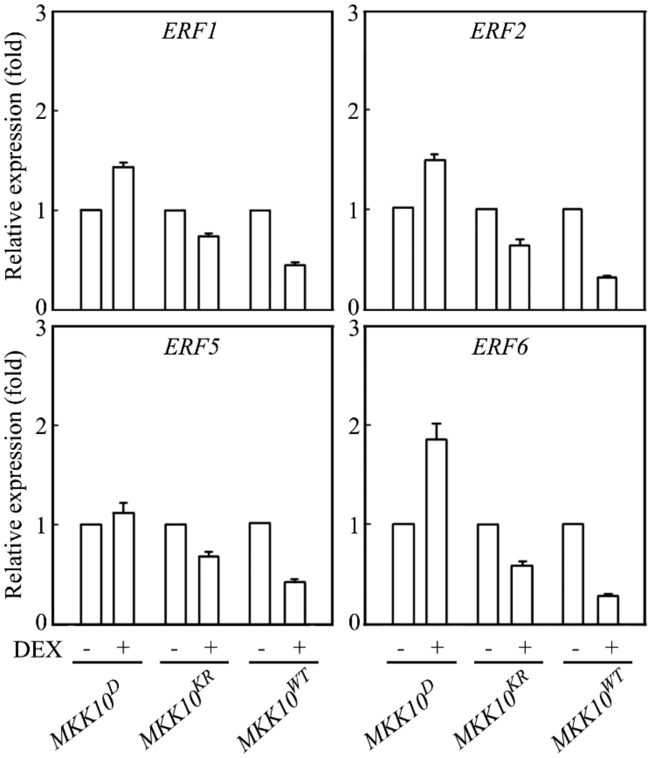
ERF1
ERF2
ERF5
ERF6
Relative expression (fold)
0
1
2
3
DEX
-
+
MKK10D
MKK10KR
MKK10WT
